# Genomic data resources of the Brain Somatic Mosaicism Network for neuropsychiatric diseases

**DOI:** 10.1038/s41597-023-02645-7

**Published:** 2023-11-20

**Authors:** McKinzie A. Garrison, Yeongjun Jang, Taejeong Bae, Adriana Cherskov, Sarah B. Emery, Liana Fasching, Attila Jones, John B. Moldovan, Cindy Molitor, Sirisha Pochareddy, Mette A. Peters, Joo Heon Shin, Yifan Wang, Xiaoxu Yang, Schahram Akbarian, Andrew Chess, Fred H. Gage, Joseph G. Gleeson, Jeffrey M. Kidd, Michael McConnell, Ryan E. Mills, John V. Moran, Peter J. Park, Nenad Sestan, Alexander E. Urban, Flora M. Vaccarino, Christopher A. Walsh, Daniel R. Weinberger, Sarah J. Wheelan, Alexej Abyzov, BSMN Consortium, Aitor Serres Amero, Danny Antaki, Dan Averbuj, Laurel Ball, Sara Bizzotto, Craig Bohrson, Rebeca Borges-Monroy, Martin Breuss, Sean Cho, Chong Chu, Changuk Chung, Isidro Cortes-Ciriano, Michael Coulter, Kenneth Daily, Caroline Dias, Alissa D’Gama, Yanmei Dou, Jennifer Erwin, Diane A. Flasch, Trenton J. Frisbie, Alon Galor, Javier Ganz, Doga Gulhan, Robert Hill, August Yue Huang, Andrew Jaffe, Alexandre Jourdon, David Juan, Sattar Khoshkhoo, Sonia Kim, Huira C. Kopera, Kenneth Y. Kwan, Minseok Kwon, Ben Langmead, Eunjung Alice Lee, Sara Linker, Irene Lobon, Michael A. Lodato, Lovelace J. Luquette, Gary Mathern, Tomas Marques-Bonet, Eduardo A. Maury, Michael Miller, Manuel Solis Moruno, Rujuta Narurkar, Apua Paquola, Reenal Pattni, Raquel Garcia Perez, Inna Povolotskaya, Patrick Reed, Rachel Rodin, Chaggai Rosenbluh, Soraya Scuderi, Maxwell Sherman, Richard Straub, Eduardo Soriano, Chen Sun, Jeremy Thorpe, Vinay Viswanadham, Meiyan Wang, Xuefang Zhao, Bo Zhou, Weichen Zhou, Zinan Zhou, Xiaowei Zhu

**Affiliations:** 1 Program in Biochemistry, Molecular and Cellular Biology, Johns Hopkins School of Medicine, Baltimore, MD 21205 USA; 2 Department of Quantitative Health Sciences, Center for Individualized Medicine, Mayo Clinic, Rochester, MN 55905 USA; 3 Department of Neuroscience, Yale University School of Medicine, New Haven, CT 06520 USA; 4 Department of Human Genetics, University of Michigan Medical School, Ann Arbor, MI 48109 USA; 5 Child Study Center, Yale University, New Haven, CT 06520 USA; 6 Department of Genetics and Genomic Sciences, Icahn School of Medicine at Mount Sinai, New York, NY 10029 USA; 7 Department of Cell, Developmental and Regenerative Biology, Icahn School of Medicine at Mount Sinai, New York, NY 10029 USA; 8 Sage Bionetworks, 2901 Third Ave., Suite 330, Seattle, WA 98121 USA; 9 Lieber Institute for Brain Development, Baltimore, MD 21205 USA; 10 Department of Neurology, Johns Hopkins School of Medicine, Baltimore, MD USA; 11 Rady Children’s Institute for Genomic Medicine, 7910 Frost St., Suite #300, San Diego, CA 92123 USA; 12 Department of Neurosciences, University of California San Diego, La Jolla, California USA; 13 Friedman Brain Institute, Icahn School of Medicine at Mount Sinai, New York, NY USA; 14 Icahn Institute for Data Science and Genomic Technologies, Icahn School of Medicine at Mount Sinai, New York, NY USA; 15 Laboratory of Genetics LOG-G, Salk Institute for Biological Studies, La Jolla, CA 92037 USA; 16 Department of Computational Medicine and Bioinformatics, University of Michigan Medical School, Ann Arbor, Michigan 48109 USA; 17 Department of Internal Medicine, University of Michigan Medical School, Ann Arbor, Michigan 48109 USA; 18 Department of Biomedical Informatics, Harvard Medical School, Boston, MA USA; 19 Department of Psychiatry and Behavioral Sciences, Stanford University School of Medicine, Stanford, California 94305 USA; 20 Department of Genetics, Stanford University School of Medicine, Stanford, California 94305 USA; 21 Division of Genetics and Genomics and Howard Hughes Medical Institute, Boston Children’s Hospital, Departments of Pediatrics and Neurology, Harvard Medical School, Boston, MA USA; 22 Department of Psychiatry and Behavioral Sciences, Johns Hopkins School of Medicine, Baltimore, MD 21205 USA; 23 McKusick Nathans Institute of Genetic Medicine, Johns Hopkins School of Medicine, Baltimore, MD USA; 24 Department of Neuroscience, Johns Hopkins School of Medicine, Baltimore, MD USA; 25 Department of Oncology, The Sidney Kimmel Comprehensive Cancer Center, Johns Hopkins University School of Medicine, Baltimore, MD USA; 32 Present Address: National Human Genome Research Institute, National Institutes of Health, 6700B Rockledge Dr, Bethesda, MD 20892 USA; 26 Institut de Biologia Evolutiva (CSIC-Universitat Pompeu Fabra), PRBB, 08003 Barcelona, Catalonia Spain; 27 Department of Neurology, Kennedy Krieger Institute, Baltimore, MD 21205 USA; 28 Department of Computer Science, Johns Hopkins University, Baltimore, MD USA; 29 Department of Molecular, Cell, and Cancer Biology, University of Massachusetts Chan Medical School, Worcester, MA 01605 USA; 30 University of California, Los Angeles, CA USA; 31 Department of Cell Biology, Physiology and Immunology, and Institute of Neurosciences, University of Barcelona, 08028 Barcelona, Spain

**Keywords:** Genetics research, Diseases of the nervous system

## Abstract

Somatic mosaicism is defined as an occurrence of two or more populations of cells having genomic sequences differing at given loci in an individual who is derived from a single zygote. It is a characteristic of multicellular organisms that plays a crucial role in normal development and disease. To study the nature and extent of somatic mosaicism in autism spectrum disorder, bipolar disorder, focal cortical dysplasia, schizophrenia, and Tourette syndrome, a multi-institutional consortium called the Brain Somatic Mosaicism Network (BSMN) was formed through the National Institute of Mental Health (NIMH). In addition to genomic data of affected and neurotypical brains, the BSMN also developed and validated a best practices somatic single nucleotide variant calling workflow through the analysis of reference brain tissue. These resources, which include >400 terabytes of data from 1087 subjects, are now available to the research community via the NIMH Data Archive (NDA) and are described here.

## Background & Summary

Somatic mosaicism is defined as an occurrence of two or more populations of cells having genomic sequences differing at given loci in an individual who is derived from a single zygote; it is a fundamental property of all humans. These genomic differences can arise in the soma at any stage in life, and the abundance of a particular genotype in a cell lineage depends on when the mutation occurred in the person’s developmental history^1,2^. The somatic mosaic mutations can be advantageous, deleterious, or neutral to the cells, and may also play a role in disease. Most commonly, cancers may arise due to somatic alterations that confer uncontrolled growth. Rare diseases have also been documented to be caused by somatic mosaic mutations, including cases in which a mutation is disease-causing when mosaic but would otherwise be lethal if it were present in the germline^3^. The role of mosaicism in common diseases is less well characterized outside of cancer, though it has been demonstrated to contribute to neuropsychiatric disease^4–9^.

The Brain Somatic Mosaicism Network (BSMN; https://bsmn.synapse.org) is a consortium formed to assess the nature and extent of somatic mosaic variation in neuropsychiatric conditions such as autism spectrum disorder (ASD), bipolar disorder (BP), focal cortical dysplasia (FCD), schizophrenia (SCZ), and Tourette syndrome (TS). Its efforts have yielded over 400 terabytes of data from a variety of different tissues and sequencing assays^3–7^. This work has led to the development of multiple computational tools and pipelines, including a best practices workflow for calling somatic variants that are mosaic^8–14^. As this computational workflow was developed, a neurotypical brain was thoroughly sequenced and validated via collective participation across the consortium. The genomic data of this brain can be used as a reference for somatic mosaic variant calling^12^. An additional neurotypical reference was sequenced across multiple tissues of various organs and regions of the brain, and is also available to the research community^14^. The data compiled for these conditions, the controls, and the reference subjects are available in the National Institute of Mental Health Data Archive (NDA) collections associated with each participating lab. Whole genome sequencing (WGS) at both conventional and high coverages, whole exome sequencing (WES), single cell sequencing, RNA sequencing (RNA-seq), and sequencing targeting somatic LINE-1 associated variants (SLAV-seq) are available to researchers with NDA access. Descriptions of the data available within the NDA repositories are available at the BSMN portal at https://bsmn.synapse.org/Explore/Data.

These data, which include a wide spectrum of genomic assays and donors, are a resource for further analyses and methods development in understanding somatic mosaicism and neuropsychiatric disease and are extensively described below.

## Methods

The BSMN consists of nine disease-specific projects, as well as a tenth consortium-wide study used to develop a somatic mosaic variant best practices workflow. Full details of how the data are structured are provided in the “Data Records” section, including links of how to access them. Below are the experimental methods for each of these collections.

### Somatic mosaicism reference data – NDA collection 2458

To produce a best practices workflow for somatic mosaic single nucleotide variant (SNV) calling, the BSMN orchestrated whole genome, whole exome, linked read, and single cell sequencing of a neurotypical reference brain (NRB) obtained from the Lieber Institute for Brain Development (LIBD). The NRB is identified in the NDA repository as both LIBD Subject 5154 and globally unique identifier NDAR_INVRT663MBL. In addition to the NRB, an experiment was performed to simulate somatic mosaicism by combining the DNA of different individuals in known quantities before sequencing. These data are available in NDA collection 2458. The methods that produced the data are described below and are thoroughly detailed in Wang *et al*.^8^. We first describe the methods and assays used to process the neurotypical reference brain before describing the experiment of combining known proportions of DNA of different individuals to simulate mosaicism.

Deep-frozen tissue of the neurotypical brain of LIBD Subject 5154, a 49-year-old white male, were extracted at a postmortem interval of approximately 30 hours. In the development of the somatic SNV workflow, each BSMN group was responsible for sample processing and SNV calling; therefore, the frozen brain samples were pulverized to uniformity prior to distribution to each lab (uniform dorsolateral prefrontal cortex; uDLPFC), with one exception in which the group received a frozen piece of the DLPFC (cDLPFC). Fibroblasts cultured from the dura mater were used as a control and were harvested following extraction to obtain consistent aliquots for each lab. Assay information is summarized in Table 1. In addition to the uDLPFC, cDLPFC, and cultured fibroblasts, sequenced samples included NeuN+ neurons, NeuN− cell fraction, cerebellum, and dura mater of LIBD 5154.

**Table 1:** Summary of the assays applied to tissues of the neurotypical somatic mosaicism reference brain in C2458.

Assay No.	Type	Tissues	DNA Extraction Kit	Library Prep Kit	Sequencing Platform	Coverage
1	WES	uDLPFC, Fibroblasts	MagAttract HMW DNA Kit	SeqCap EZ Exome Probes v3.0	Illumina HiSeqX	350 - 435X
2		uDLPFC, Fibroblasts	Qiagen Maxiprep Kit	Agilent SureSelect X7 Human All Exon v5 Kit	Illumina HiSeqX 2500	
3	WGS	cDLPFC	DNeasy Blood and Tissue Kit	NEBNext Ultra II DNA Library Prep Kit	Illumina HiSeq. 4000	85 - 245X
4		uDLPFC, Fibroblasts, cerebellum, dura mater, NeuN+, NeuN−	DNeasy Blood and Tissue Kit	TruSeq DNA PCR-Free Library Prep Kit	Illumina HiSeqX	
5		uDLPFC	QIAamp DNA Mini Kit with phenol chloroform and isopropanol workup	TruSeq DNA PCR-Free Library Prep Kit	Illumina HiSeqX Ten	
6		uDLPFC, Fibroblasts, uDLPFC NeuN+, uDLPFC NeuN−	DNeasy Blood and Tissue Kit	TruSeq DNA PCR-Free Library Prep Kit	Illumina HiSeq. 2000	
7	LR	uDLPFC, Fibroblasts	MagAttract HMW DNA Kit	Submission to HudsonAlpha Discovery	10X Genomics	~70X
8	sc	uDLPFC NeuN+	(1) REPLI-g Single Cell Kit & DNeasy Blood and Tissue Kit	(1) TruSeq DNA PCR-Free Library Prep Kit	Illumina HiSeqX	~30X
		uDLPFC NeuN+	(2) REPLI-g Single Cell Kit	(2) S1 Maker 10 kb High Pass Protocol on BluePippin	10X Genomics & Illumina HiSeqX	

Four assays were applied to various tissues and cells (WES = whole exome sequencing, WGS = whole genome sequencing, LR = linked read, sc = single cell sequencing). Tissues included uniformly pulverized dorsolateral prefrontal cortex (uDLPFC), frozen chunk of dorsolateral prefrontal cortex (cDLPFC), fibroblasts cultured from the dura mater, NeuN+ neurons, NeuN− cell fraction, the cerebellum, and the dura mater.

Sample processing for each sequencing assay was performed by a different participating working group. Manufacturer’s protocols were followed for all DNA extraction and library preparation kits unless a customization was specified.

WES was performed with two methods on the uDLPFC and fibroblast samples. The first method used a MagAttract HMW DNA kit for DNA extraction, and DNA length was checked by either standard or pulse field gel electrophoresis. The genomic DNA was sheared to 350 bp and 0.65X SPRIselect beads were used for gel-free size selection. The exome was captured and amplified using SeqCap EZ Exome Probes v3.0 followed by sequencing on an Illumina HiSeqX platform. The second method used Qiagen Maxiprep kits for DNA extraction, with target enrichment using an Agilent SureSelect XT Human All Exon v0.5 kit. Sequencing was performed on an Illumina HiSeqX 2500 machine.

Four WGS procedures were applied to different NRB regions and fractions (Table 1). One included DNA extraction via the QIAamp DNA Mini kit with phenol chloroform and isopropanol workup on a uDLPFC sample. Seven libraries were prepared with the TruSeq DNA PCR-free kit, ≥30X coverage was obtained for each library on an Illumina HiSeqX Ten, and the results were combined for a final coverage of ≥210X. The second method used a frozen chunk of the DLPFC (cDLPFC) rather than the tissue that had been pulverized prior to distribution. The external tissue was removed before the piece was minced and its DNA was extracted with the DNeasy Blood and Tissue kit. Sequencing was performed with an Illumina HiSeq. 4000 on a library produced with the NEBNext Ultra II DNA library prep kit.

The final two WGS assays used essentially identical procedures, though in addition to the uDLPFC brain tissue, fibroblasts, and NeuN+ /NeuN− fractions, one of the two assays also used processed pulverized cerebellum and dura mater tissue of the NRB. Isolation of the NeuN+ and NeuN− samples involved size fractionation with sucrose density gradient centrifugation at 25,000 rpm, 4 °C for 1 hr with 0.7 g of the uDLPFC sample. Centrifugation was followed up with FACS on a BD FACSAria III machine after anti-NeuN-488 Millipore MAB377X antibody was incubated with the sample in PBS and 0.1% BSA at a dilution ratio of 1:1000. DNA extraction and library preparation was performed using the DNeasy Blood and Tissue kit with its RNase A step and the TruSeq DNA PCR-free library prep kit, respectively. Sequencing was performed on Illumina HiSeqX and HiSeq. 2000 machines.

Linked read sequencing was performed on the uDLPFC and fibroblast samples. The MagAttract HMW DNA kit was used for DNA extraction, DNA length was checked by standard or pulse field gel electrophoresis, and 10X Genomics linked read sequencing services were procured for 1–5 µg of DNA from the HudsonAlpha Genome Sequencing Center. Alignment and phasing were performed with Long Ranger v2.2.

Finally, single cell sequencing was performed on an NeuN+ fraction, isolated in the same manner and from the same tissue type as what has been previously described for the bulk WGS assays. Following sorting, 95 NeuN+ nuclei were distributed to separate wells in a 96-well plate before being subjected to multiple displacement amplification (MDA) with the Qiagen REPLI-g single cell kit. DNA extraction was performed with the DNeasy Blood and Tissue kit. Two major quality control filters were applied to the MDA reactions to choose nuclei for single cell sequencing. The first quality control measure required that four loci be successfully amplified for each well. About 70.5% of the reactions passed. The second quality control filter involved assessing the samples using low coverage WGS to recheck the loci. Twelve samples passed the second filter.

Ultimately, seven of twelve MDA reactions that passed the quality control checks were sequenced to 30X coverage. Their DNA libraries were produced using the Illumina TruSeq DNA PCR-Free kit and sequencing was performed on an Illumina HiSeqX platform. The remaining five wells underwent size selection purification with the S1 Maker 10 bp High Pass Protocol for high molecular weight fragments greater than 10 kb. These libraries were PCR amplified, diluted, and then sequenced on an Illumina HiSeqX sequencer^8^.

In a different approach for validating methods of calling mosaic variation, known proportions of genomic DNA derived from the unrelated grandparents of CEPH/Utah pedigree 1463, a family containing the well-characterized subject NA12878, were mixed and sequenced. Phenol chloroform extraction was performed on pedigree 1463 lymphoblastoid cell lines GM12889, GM12891, GM12890, and GM12892 and aliquots of the resulting DNA were mixed in three different proportions, with one sample being processed without mixing (Table 2). DNA libraries were produced using the Illumina PCR-free TruSeq DNA Library Prep kit for 1 µg of mixed DNA, and library quantification was checked using qPCR and the KAPA Library Quantification kit before WGS was conducted on an Illumina HiSeq X instrument^8^. Alignment files were produced using the uniform processing pipeline described in the Data Records section.

**Table 2:** Overview of the samples with mixed cell lines.

gDNA Mixtures	Sample Proportions			
	GM12889	GM12891	GM12890	GM12892
**Mix 1**	1	2	4	18
**Mix 2**	1	2	4	43
**Mix 3A**	0	0	0	1
**Mix 3B**	3	6	12	79

Genomic DNA extracted from lymphoblastoid cell lines of four unrelated individuals was mixed in different proportions before 1 microgram of the resulting mixture was processed and sequenced. For example, Mix 1 is comprised of a 1:2:4:18 ratio. These data are available in C2458.

### Somatic mosaicism reference data – NDA collection 2968

A unique dataset for the study of somatic mosaicism is available in NDA collection 2968. Twenty-five tissue samples from the brain, heart, liver, and kidneys were obtained from a neurotypical 70-year-old Caucasian female (UCSD-19-110) within a 24-hr postmortem interval. Samples from case number UCSD-19-110 were obtained through the UC San Diego Anatomical Material Program. Samples obtained from human cadavers are exempt from oversight via a state regulated IRB. Approval and study oversight were instead performed by the UC San Diego Anatomical Materials Committee (approval no. 106135). Note that the phenotype of this subject is marked as global geriatric decline (GGD). The prefrontal, frontal, parietal, occipital, and temporal lobes of both hemispheres of the brain were dissected, and thirteen 8 mm diameter 1 cm thick punches were collected. Two punches also were collected from the heart, liver, cerebellum, and each kidney.

Tissue punches of the cortical lobes were separated into “Sml” and “Lrg” categories. Each “Sml” sample was taken from a centralized punch of its respective lobe. The remaining punches of each site were homogenized followed by nuclear extraction and were marked as “Lrg.” Note that these categories are included in their related file names in the data repository.

Each category of tissue underwent a series of processing steps. The Lrg punches were used for nuclear preparations, where they were homogenized by grinding at −196 °C, motorized homogenization (1% formaldehyde in PBS), rocking at room temperature for 10 min, and then the addition of 0.125 M glycine with another 5 min of rocking. After centrifugation, the samples were kept at 0 °C, washed twice (10 mM Tris-HCl pH 8.0, 1 mM EDTA, 5 mM MgCl_2_, 0.1 M sucrose, 0.5% Triton X-100), centrifuged (1100RCF, 5 min, 4 °C), resuspended in 5 ml washing buffer, and then dounced five times. Homogenates were kept at 0 °C for 30 min before being dounced twenty times. Particulate matter was removed (70 µm sieve opening), and further purification was performed with a sucrose cushion (1.2 M sucrose, 1 M Tris-HCl pH 8.0, 1 mM MgCl_2_, 0.1 M DTT; 3200RCF, 30 min). The resulting nuclear fractions were stored at −80 °C.

These nuclear fraction homogenates (Lrg), homogenized central tissue punch of lobar tissue (Sml), and punches of the cerebellum, left and right kidneys, liver, and heart were used for high depth (300X) WGS. DNA extraction and purification were performed using the AllPrep DNA/RNA Mini kit (Qiagen) according to protocol for −80 °C stabilized tissue. Library preparation was performed using the KAPA HyperPrep PCR-Free Library Prep kit. The Agilent DNA High Sensitivity NGS Fragment Analysis and KAPA Library Quantification kits were used for quality control and quantification. Sequencing was performed on an Illumina NovaSeq. 6000 platform. BAM alignment files were outputted by the Illumina DRAGEN Bio-IT platform. They were converted to fastq files (PICARD v2.20.7) to be aligned to GRCh37d5 with bwa mem (v0.7.17) with parameters set to use soft clipping for supplementary alignments^15–17^. The alignment files were sorted before indel regions were realigned with GATK (v3.8-1) and recalibrated according to best practices^18^. Coverage for the WGS samples, marked under UCSD-19-110, is shown in Supplementary Fig. 1). HaplotypeCaller was used to find germline variants^14,19^.

Nuclear isolation buffer (0.25 M sucrose, 25 mM KCl, 5 mM MgCl_2_, 10 mM Tris pH 7.5, 100 mM DTT, 0.1% Triton X-100; 0 °C) and douncing were used to produce a homogenate of tissue from the left temporal lobe for single nuclei sorting and MPAS. The resulting sample was rocked and washed similarly to the nuclear extraction described for the Lrg samples, and it was stained using a 30-min incubation with Millipore Sigma NeuN Alexa Fluor 488 with a dilution factor of 1:2500. DAPI staining was performed with 0.5 µg/mL, and the nuclei were sorted on a Becton-Dickinson BD InFlux Cytometer into a 96-well plate. Whole genome amplification was performed with a REPLI-g Single Cell kit^14^.

In addition to WGS, the nuclear fractions were also sorted for cell origin (neurons, oligodendrocytes, astrocytes, and microglia) for massive parallel amplicon sequencing (7000X) targeting variant sites. This protocol involved washing the pellets twice (HBSS without Mg^2+^ and Ca^2+^, 5% BSA, 1 mM EDTA) for antibody staining and FANS. Incubation occurred overnight at 4 °C with three unconjugated antibodies (Abcam #ab31940 1:1000 dilution of TBR1, Abcam #ab1091986 1:1000 dilution of OLIG2, Abcam #ab2199883 1:500 dilution of LHX2) and two conjugated antibodies (Millipore Sigma MAB377 1:2500 dilution of NeuN Alexa Fluor 488, BioLegend #658004 dilution of 1:100 PV.1 Alexa Fluor 647). The unconjugated antibodies required additional incubation of 30 min with either Thermo Fisher Scientific #A21244 1:4000 dilution of goat αRabbit Alexa 647 (TBR1 and LHX2) or Thermo Fisher Scientific #A32732 1:4000 goat αRabbit Alexa 555 (OLIG2). DAPI staining was applied before sorting on a Beckman Coulter MoFlo Astrio EQ sorter or a Becton-Dickson BD InFlux Cytometer.

MPAS was performed on the single nuclei temporal cortex samples, the sorted cell fractions, the Sml homogenates, and the Lrg nuclear fractions. AmpliSeq software was used to design primers for the AmpliSeq Custom DNA Panel. The AmpliSeq Library PLUS kit was used to produce DNA libraries. Indexing was performed with AmpliSeq CD Indexes. Sequencing was completed with an Illumina NovaSeq. 6000. The fastq files were aligned to GRCh37d5 with bwa mem (v0.7.17), and recalibration was performed with GATK best practices (v3.8-1)^14–18^.

### Autism spectrum disorder – NDA collection 2962

NDA collection 2962 utilized de-identified postmortem human brain tissues that were collected by the University of Maryland Brain and Tissue Bank (an NIH NeuroBioBank; institutional review board (IRB) no. HP-00042077), the Autism BrainNet (McLean IRB nos. 2006-P-0001161 and 2006-P-0001862; Western IRB no. 20141029), and the LIBD (Maryland Dept of Health and Mental Hygiene IRB no. 12–14; Western IRB no. 20111080) following provision of informed consent. The secondary use of these tissues for genomic studies was approved by the Boston Children’s Hospital Institutional Review Board (protocol S07-02-0087).

The majority of the data of collection 2962 is composed of high depth whole genome sequencing (≥210X coverage) of either the DLPFC or the prefrontal cortex tissue of 59 ASD-affected and 15 unaffected subjects. Additional tissues that underwent WGS include two occipital lobe samples of controls and the saliva of parents of two affected individuals. DNA extraction was performed with the QIAamp DNA Mini kit lysis buffer, phenol chloroform extraction, and isopropanol workup.

One of two methods was applied to obtain high depth WGS data. The first method involved sequencing of seven libraries per sample produced by the Illumina TruSeq DNA PCR-Free library prep kit on an Illumina HiSeqX Ten platform to yield a combined coverage of ≥210X. The second method used sequencing of a single library produced with the Illumina TruSeq Nano DNA library kit on an Illumina HiSeqX Ten for 200X coverage in one run (Supp. Fig. 1)^5^.

The data were aligned to GRCh37d5 using bwa mem 0.7.8^15–17^. Variant calling was performed with Mutect-Panel-of-Normals according to GATK 3.5 best practices^18,20^. Point mutation variants identified within segmental duplications and non-diploid regions were filtered from the results. The Genome Aggregation Database was used to set a threshold for filtering variants at a maximum population minor allele frequency above 1 × 10^−5^ ^21^ Population polymorphisms were also removed^22^. Variants outside a variant allele frequency between 0.02 to 0.40 were also filtered. MosaicForecast was used for phasing and identifying postzygotic variants^9^.

### Autism spectrum disorder – NDA collection 2960

Brain tissues of the DLPFC, cerebellum, inferior temporal cortex, primary visual cortex, and striatum were extracted from 40 individuals (n = 14 ASD-affected, n = 5 ASD suspected, and n = 21 unaffected controls) to undergo RNA sequencing. These samples were obtained from the NIH NeuroBioBank, the University of Maryland Brain and Tissue Bank, and Yale University, using the consent procedures of each respective institution. All samples were obtained postmortem. Study approval was provided through the Yale University Human Research Protection Program (IRB no. 0605001466). Tissue dissection procedures are described in Kang *et al*., 2011^23^. The total RNA was isolated from the samples using the mirVana kit (Ambion). Deviations from the manufacturer’s protocols included each tissue sample being pulverized with liquid nitrogen in a pre-chilled mortar and pestle prior to being transferred to a chilled safe-lock microcentrifuge tube. An equal mass of chilled stainless steel beads (Next Advance, catalog #SSB14B) relative to the mass of the tissue and an equivalent volume of lysis/binding buffer were added. The tissue was homogenized for one minute in a Bullet Blender (Next Advance) before being incubated at 37 °C for one minute. Another nine volumes of the lysis/binding buffer were added and were homogenized for one minute before incubation at 37 °C for two minutes. The miRNA Homogenate Additive was added at one-tenth the volume of the lysate, and extraction proceeded according to the manufacturer’s protocol. The RNA samples were then treated with DNase using the TURBO DNA-free kit (Ambion/Life Technologies) and the RNA integrity was measured using Agilent 2200 TapeStation System.

Barcoded libraries were produced with 5 ng of RNA using TruSeq Stranded Total RNA HT Sample Prep Kit with Ribo-Zero Gold kit according to the manufacturer’s protocol (Illumina). Paired-end sequencing was performed on Illumina HiSeq. 4000 platforms (100 bp × 2) at the Yale Center for Genome Analysis.

### Bipolar disorder – NDA collection 2964

Four cohorts were studied in the BP context: (1) samples from the NIMH Repository and Genomics Resource (NRGR) (n = 200 exomes), (2) the Stanley Medical Research Institute (SMRI) Array Collection (n = 35 bipolar disorder, n = 35 schizophrenia, n = 35 neurotypical control exomes), (3) postmortem brains from LIBD (n = 19 bipolar disorder, n = 10 control 10X linked read WGS), and (4) samples from Sheppard Pratt (n = 43 bipolar-depressed, n = 73 bipolar-mania, n = 15 bipolar marked neither as mania nor depression, n = 8 major depression, n = 124 controls). Samples obtained from each biorepository obtained consent according to the tissue banks’ internal procedures. Brain and blood samples were studied at the Kennedy Krieger Institute with the approval of the Johns Hopkins University IRB no. NA_00001324.

Samples from a set of 30 pedigrees were obtained from the NRGR (https://www.nimhgenetics.org/). Each family contained a proband under the age of 18 as well as a trio of mother, father, and child. Genomic DNA was derived from either lymphoblastoid cell lines (n = 194) or whole blood (n = 6). These samples were genotyped on an Illumina Infinium SNP array (GSA MG v2, 766,221 SNPs; Macrogen, Inc.). After quality control was completed, n = 199 samples were analyzed. Genotyping results were summarized in.ped and.map files and further quality control was performed with SNPduo and PLINK software^24,25^. Whole exome sequencing was performed on an Illumina HiSeq 2500 platform (Macrogen, Inc.) at 200X average depth of coverage. After further quality control was performed with FastQC, germline and mosaic variant calling were performed with a Sentieon workflow on Google Cloud Platform^26^.

Whole exome sequencing was also performed on prefrontal cortex and hippocampus samples obtained from the SMRI Array Collection. Frozen tissue was pulverized before genomic DNA was isolated with the DNeasy Blood and Tissue kit. Target enrichment and DNA library preparation were performed with the Agilent SureSelectXT v5 and DNA Library kits. Sequencing was conducted on an Illumina HiSeq. 4000 platform. The bwa mem (0.7.15) tool was used to align the sequencing data to GRCh37d5^15–17^. Germline variants were called using GATK HaplotypeCaller (3.8.0), XHMM (1.0), and CNVkit (0.9.2)0^19,27,28^. A panel of somatic variant calls representing normal samples was generated for all groups (GATK 4.0.1.0) and somatic SNVs were called using reciprocal brain regions with MosaicHunter (1.0) and Mutect2 (4.0.1.0)^29,30^. Mutect2 was also used for single samples.

Strict filters were applied to the SMRI data. These included the removal of population polymorphisms with allele frequencies greater than 0.001 in gnomAD (2.1.0)^21^. Any somatic SNV calls within repetitive or low complexity intervals were removed, as were any calls with less than five supporting reads. If any SNV calls were within 500 bp of germline calls or were near one another by 1,000 bp they were also filtered. Variants outside the 1000 Genomes Phase 3 mappability mask were removed^22^. Variants were also removed if the alternate allele was supported in the Platinum Genomes 200X WGS NA12878 data^31^.

The Sheppard Pratt cohort consists of n = 43 bipolar-depressed, n = 73 bipolar-mania, n = 15 bipolar marked as neither mania nor depression, and n = 8 major depression affected individuals, as well as n = 124 subjects unaffected by bipolar disorder or depression. Blood samples were used to establish lymphoblastoid cell lines before genomic DNA was purified using a DNeasy Blood and Tissue kit. Target enrichment and DNA library preparation were performed with the Illumina Nextera library kit followed by hybrid capture using Illumina rapid capture enrichment of the 37 Mb target. Whole exome sequencing was conducted at the Broad Institute on Illumina HiSeqX instruments. Sequencing was run until hybrid selection libraries met or exceeded 85% of targets at 20X with 150 bp paired-end reads, comparable to approximately 55X mean coverage (Supp. Fig. 2).

Data delivery per sample included demultiplexing and aggregation into a BAM file, with reads aligned using bwa to the human genome build 38 (GRCh38), which was processed through a pipeline based on the PICARD suite of software tools^15,16^. Single nucleotide polymorphism and insertions/deletions were jointly called across all samples using the GATK HaplotypeCaller package (4.0.10) to produce a version 4.2 variant callset file (VCF)^19^. Variant call accuracy was estimated using the GATK Variant Quality Score Recalibration (VQSR) approach^18,19^.

For sequencing with 10X Genomics linked read technology, samples from n = 30 postmortem individuals were obtained from the LIBD, consisting of individuals diagnosed with BP (n = 20) or neurotypical controls (n = 10). Samples were matched for sex (n = 7/20 females with BP, n = 4/10 female controls), race (3/20 African Americans in bipolar cohort), age at death (average 44.1 years for bipolar cohort), and postmortem interval (range 9 to 54.5 hours, mean 29.6 hours for bipolar cohort). The cause of death was suicide in n = 12 BP cases but no control cases. The DLPFC was dissected to obtain approximately 100 mg per sample. High molecular weight genomic DNA was obtained (Qiagen MagAttract protocol), the integrity was confirmed (TapeStation), and libraries were created. These samples were sequenced (Macrogen Clinical Laboratories, Inc.) to 60X average depth of coverage. Data analysis was performed using Long Ranger software (10X Genomics). One sample (Br1470) failed quality control yielding data from n = 19 bipolar disorder cases. Mosaic variants were assessed using Samovar using custom filtering scripts^32^.

### Focal cortical dysplasia – NDA collection 2968

Focal cortical dysplasia (FCD) samples were obtained with approval by UC San Diego IRB no. 140028 and processed using whole exome sequencing. Informed consent was obtained from all participants or their legal guardians at the time of enrollment. Diagnostic criteria for the FCD samples included detection of malformations of cerebral cortical development due to altered neuronal proliferation or migration via neuroimaging or an abnormal electroencephalogram (EEG) from focal seizures without cortical malformations. After surgery, a pathological examination was performed on the specimen to determine the FCD subtype following established criteria^33^.

Biopsies of brain samples were predetermined by preoperative scans and checked during the surgical procedure with the aid of a neuronavigation system. The biopsied tissue was then fractionated into two parts; one was sent for anatomopathological study and another for genetic analysis. A venous blood sample, saliva sample, or both were also collected during surgery.

Brain tissue, peripheral blood, and saliva-derived DNA samples underwent exome capture. To this end, DNA was extracted using a QIAamp DNA Mini kit (Qiagen) or DNA Purification kit (Promega). Libraries were prepared from 250 ng of genomic DNA, and exonic regions were captured by using the Illumina Nextera Rapid Capture Exome kit or Agilent SureSelect (Human All Exon 50 Mb) and SureSelectXT Exome Capture kits. Paired-end reads of either 100 bp or 125 bp in size were sequenced on an Illumina HiSeq 2500 sequencer with V1 or V4 kits. Two libraries from each brain sample were sequenced to achieve a coverage of approximately 300X and one library was sequenced for the blood or saliva samples to reach 100X coverage (Supp. Fig. 2).

The data processing procedure to yield CRAM files was identical to the BSMN common processing pipeline described for the NRB data. Raw fastq files were also uploaded to the NDA collection.

### Schizophrenia – NDA collection 2967

NDA collections 2967, 2966, and 2963 contain some subjects that are shared between repositories. All samples were obtained postmortem and were de-identified. Shared subject samples were obtained through the LIBD Human Brain and Tissue Repository. Donations to the LIBD were collected through the Office of the Chief Medical Examiner of the State of Maryland under the Maryland Department of Health’s IRB protocol #12–24. Audiotaped and witnessed informed consent was obtained from the legal next-of-kin for every case at the time of autopsy.

The LIBD Autopsy telephone screening was done at time of donation with the legal next-of-kin and consisted of 39 items about the donor’s medical, social, psychiatric, substance use, and treatment history. Retrospective clinical diagnostic reviews were conducted for every brain donor to include data from: autopsy reports, toxicology testing, forensic investigations, neuropathological examinations, telephone screening, and psychiatric/substance abuse treatment record reviews and/or supplemental family informant interviews. All data was compiled and summarized in a detailed psychiatric narrative summary and was reviewed independently by two board-certified psychiatrists to determine lifetime psychiatric diagnoses according to DSM-5. Non-psychiatric control donors were excluded if they had acute drug and alcohol intoxication/use at time of death.

Additional samples were obtained from the NICHD Brain and Tissue Bank for Developmental Disorders at the University of Maryland and the University of Virginia Biorepository (NDA collection 2963). Each tissue bank followed their respective procedures for consent, donation, and obtaining and archiving samples.

The LIBD performed a series of matching procedures on case and control brain samples to maximize the likelihood of finding mosaicism in the brain associated with a diagnosis of SCZ by matching on sex, ancestry, and age at death. This selection process included choosing young subjects with an early age of onset, controlling for various confounders that compromise molecular studies of the postmortem human brain, controlling for tissue quality, and excluding individuals with evidence of prior recurrent CNVs associated with SCZ. Table 3 summarizes the dataset generated from the LIBD as NDA collection 2967.

**Table 3:** Sequencing assay and method information, coverage, and brain regions of collection 2967.

Assay	Depth	Region	Num.	Kits	Sequencer	Enrichment
WGS	30x	CB	46	Illumina TruSeq PCR Free kit	HiSeq X	Bulk tissue
		Dura	62			
	90x	DLPFC, HIPPO	40	Illumina TruSeq Nano kit	NovaSeq	Bulk tissue
	90x	DLPFC	87	Illumina TruSeq Nano kit	HiSeq X	Prime Flow RNA Assay based enrichment (Excitatory, Inhibitory, Oligos, and Astrocyte/Microglia/Epithelial)
RNA-seq	80–100 M reads	DLPFC, HIPPO	89	Illumina TruSeq Stranded Total RNA Library Prep Gold	HiSeq. 3000	Bulk tissue

The “Num.” column describes number of samples. In total, 108 unique subjects underwent sequencing. Samples were obtained from the DLPFC (dorsolateral prefrontal cortex), HIPPO (hippocampus), CB (cerebellum), and Dura (dura mater), and underwent WGS or RNA-seq.

Samples were processed for PrimeFlow RNA sorting (Thermo Fisher). One to 1.5 g of frozen human postmortem brain tissue from the DLPFC was obtained for each sample and split into aliquots of approximately 500 mg. Each 500 mg tissue sample was placed in a chilled dounce homogenizer containing 5 mL of chilled lysis buffer (0.32 M sucrose, 3 mM magnesium acetate, 5 mM calcium chloride, 0.1 mM EDTA, 10 mM Tris HCl, ph 8.0, 0.10% Triton X-100) and thawed for 5 min on ice. Samples were then dounced 50 times each with the tight pestle, and an additional 5 mL of chilled lysis buffer was added to the solution. The tissue lysate was then layered on top of 18 mL chilled sucrose buffer (1.8 M sucrose, 3 mM magnesium acetate, 10 mM Tris HCl pH 8.0) in a 38.5 mL ultracentrifuge tube (Cat. No. 344058, Beckman Coulter) to form a gradient. Samples were ultracentrifuged using a SW32 Ti rotor at 139,800RCF (28,600RPM) for 3hrs at 4 °C.

The PrimeFlow RNA Assay was performed using the assay kit from Thermo Fisher Scientific (Carlsbad, CA) according to the manufacturer’s protocol, with some modifications. We optimized the PrimeFlow RNA approach to capture nuclei from specific cell types of interest using RNA labeling followed by FANS. We developed our own computational pipeline and designed and tested custom probes for neuronal nuclei (SNAP25), as well as for specific inhibitory (GAD1) and excitatory (SLC17A7, aka VGLUT1) neuronal subclasses, oligodendrocytes (MBP) and astrocytes (GFAP). Briefly, purified nuclei were fixed and permeabilized in Fixation Buffer 1 on ice for 30 min, followed by fixation in Fixation Buffer 2 at room temperature in the dark for 1 hr. To detect nuclear mRNA targets, fluorophore-tagged target probes were first diluted and hybridized to the targets at 40 °C. Signal amplification was then carried out through the hybridization of PreAmplifier Mix and then Amplifier Mix, each at 40 °C for 1 hr and stained with DAPI (final concentration of 1 µM) for 10 min on ice in the dark. Data were acquired on a MoFlow XDP Cell Sorter (Beckman Coulter, Indianapolis, IN) using appropriate laser lines and filters (Type 1, AlexaFluor 647; Type 4, AlexaFluor 488) and analyzed using FlowJo software (TreeStar, Inc., Ashland, OR). After nuclei sorting and collection, DNA and RNA were extracted using the AllPrep DNA/RNA FFPE kit (Qiagen, Germantown, MD) following the manufacturer’s protocol.

DNA libraries were prepared using either an Illumina TruSeq PCR Free kit or a Nano DNA kit based on multiple QC metrics. Each library was sequenced at 30X or 90X coverage at Psomagen using a HiSeq X or NovaSeq. 6000 S4 flowcell with 150 bp paired-end reads (Supp. Fig. 1). DNA input for library preparation was 300 ng for bulk WGS and 30 ng DNA from Prime Flow RNA Assay WGS.

All WGS data were uniformly processed through the BSMN uniform processing pipeline to prepare aligned BAM files. Reads from fastq files were aligned to the human reference genome GRCh37d5 using bwa (v3.7.16a), sorted per each read group, and merged into a single BAM file with sambamba (v0.6.7)^15–17,34^. The merged BAM files were marked for duplicate reads using PICARD (v2.12.1). Indel realignment and base quality recalibration was performed with GATK (v3.7-0), resulting in the final uniformed processed BAM files^18^. To call structural variants (SVs) based on these BAM files, Parliament2 was run on the DNAnexus platform, a suite that includes a combination of SV callers (Breakdancer, Breakseq. 2, CNVnator, Delly2, Manta, and Lumpy), SVTyper was used in genotyping, and Survivor was used to merge the higher specificity consensus calls^35–37^.

A total of 89 samples were used in RNA sequencing assays in two batches, with preprocessing and quality assessments from 45 unique individuals’ DLPFC and hippocampus tissues. One hippocampus sample failed. Total RNA was extracted from brain tissue using the RNeasy kit (Qiagen). Paired-end 100 bp reads were generated with a targeted coverage of 80–100 million sequencing reads per sample from libraries prepared with Illumina TruSeq stranded Ribo-zero Gold kit. Our preprocessing pipeline includes quantified QC metrics calculated with FastQC (v.0.11.5), alignment with HISAT2 (v.2.0.4), counting reads for genomic features using featureCounts (subread) (v.1.5.0-p3), and quantification of ERCC synthetic transcript spike-ins using kallisto (v.0.43.0)^38–40^. Following completion of the preprocessing pipeline, the following in-depth QC checks on samples that failed one or more of the following tests were performed: alignment rate lower than 80%, low genotype correlation between samples labeled from the same brain, or ambiguous brain region identity score based on the expression of top genes that differentiate the two regions. All remaining samples passed additional technical QC checks for mitochondrial mapping rates, ribosomal RNA mapping rates, adapter content, and expected ERCC spike-in concentrations.

### Schizophrenia – NDA collection 2966

Pulverized frozen DLPFC and hippocampal (HIPPO) brain tissue samples from 20 deceased individuals (n = 10 SCZ-affected individuals, n = 10 unaffected controls marked as N) were obtained from the LIBD. Note that all of these subjects were also present in NDA collection 2967 and some were shared with collection 2963 but underwent different sequencing workups. Each of the SCZ and control samples were matched to one another based on gender, age, and ancestry, resulting in 10 matched pairs. Dural fibroblasts (FIBRO) were received for 16 of the 20 subjects (8 matched pairs). Dural fibroblasts were cultured for 3–10 weeks and passaged when they reached ~85–95% confluence using the following growth medium: DMEM supplemented with 10% fetal bovine serum (FBS), 2% Glutamax, and a 1% Antibiotic, Antimycotic solution, which were all obtained from Gibco/Thermo Fisher (Waltham, MA).

The MagAttract High Molecular Weight DNA Kit (Qiagen, Germantown, MD) was used to isolate genomic DNA (gDNA) from the DLPFC and HIPPO of the 10 matched pairs (LIBD99-N, LIBD82-SZ; LIBD75-N, LIBD109-SZ; LIBD83-N, LIBD98-SZ; LIBD101-N, LIBD107-SZ; LIBD104-N, LIBD110-SZ; LIBD78-N, LIBD76-SZ; LIBD96-N, LIBD120-SZ; LIBD122-N, LIBD123-SZ; LIBD87-N, LIBD80-SZ; LIBD77-N, LIBD100-SZ) and from cultured FIBRO derived from 8 of the SCZ/N samples (LIBD99-N, LIBD82-SZ; LIBD83-N, LIBD98-SZ; LIBD101-N, LIBD107-SZ; LIBD104-N, LIBD110-SZ; LIBD78-N, LIBD76-SZ; LIBD96-N, LIBD120-SZ; LIBD122-N, LIBD123-SZ; LIBD87-N, LIBD80-SZ). To verify the integrity of the high molecular weight DNA, we performed either 0.4% agarose gel electrophoresis or 1% agarose pulse field gel electrophoresis (using the following conditions: 0.5x Tris-buffered EDTA, pH 8.3 [TBE] at 6 V/cm and a 1200 angle with an initial switch time of 1 s and final switch time of 6 s for 16 hr). Note that the LIBD identifiers are relevant to file names.

Whole exome sequencing was performed on each of the above DNA samples as described in the section for the neurotypical reference brain. Briefly, duplicate genomic DNA libraries were prepared in the following manner: (1) genomic DNA (75–200 ng) from each of the above samples was sheared to ~350 bp on a Covaris M220 Focused-ultrasonicator (Covaris, Woburn, MA) using microTUBE-50 and manufacturer’s recommended settings; (2) sheared DNA was purified with 1X SPRIselect beads (Beckman Coulter, Pasadena, CA). Then, the NEBNext Ultra DNA Library Prep Kit for Illumina (New England Biolabs, Ipswich, MA) was used for end repair, dA-tailing, and to ligate Nextflex adapters (Perkin Elmer, Waltham, MA) onto the sheared genomic DNA. After ligation, reactions were purified with 0.65X SPRIselect beads (Beckman Coulter) and PCR enrichment of adapter-ligated DNA was performed for 10–14 cycles using NEBNext Ultra DNA Library Prep Kit (New England Biolabs); and (3) libraries were purified with 0.65X SPRIselect beads (Beckman Coulter) and quantified using a Qubit dsDNA HS Assay Kit (Thermo Fisher Scientific, Carlsbad, CA). A 50 ng aliquot of the library was saved as a qPCR control to assess capture efficiency after exome target enrichment. The remaining 400–800 ng was used for exome target enrichment experiments.

Exome target enrichment was performed using SeqCap EZ Exome Probes v3.0 (Roche Sequencing Solutions, Pleasanton, CA) according to the manufacturer’s protocols. We used a 72-hr hybridization incubation period to capture the DNA and 12–16 cycles of post-capture ligation-mediated PCR to amplify the exome enriched DNA. A Qubit dsDNA HS Assay Kit was used to quantify the captured DNA. The exome target enrichment was calculated by determining the abundance of the exome targets in the post-capture library relative to the abundance of the exome targets in the pre-capture library as specified in the protocols in SEqCap_EZ_UGuide_v5.4. Each individual library then was subjected to quality control verification and sequenced on a single lane of a HiSeq X series sequencer at Novogene Corporation (Davis, CA). Coverage is shown in Supp. Fig. 2.

The whole exome sequencing data was uniformly processed using the BSMN uniform processing pipeline^8^. For each sample, the reads from FASTQ files were aligned to the human reference genome GRCh37d5 (ftp://ftp-trace.ncbi.nih.gov/1000genomes/ftp/technical/reference/phase2_reference_assembly_sequence/hs37d5.fa.gz) using bwa (version 3.7.16a), sorted per each read group, and merged into a single BAM file with sambamba (version 0.6.7)0^15,17,34^. The merged BAM files were marked for duplicate reads using PICARD (v2.12.1). Then, we performed indel realignment and base quality recalibration using GATK (v3.7–0), resulting in the final uniformed processed BAM files^18^.

Linked read sequencing was also performed on the samples. An aliquot of gDNA (1–5 ug) from the DLPFC samples from the 10 neurotypical/SCZ matched pairs was used to generate 10X Genomics (Pleasanton, CA) linked read sequencing data. The samples were sequenced on the Illumina NovaSeq. 6000 platform at HudsonAlpha Genome Sequencing Center (Huntsville, AL). The resultant reads were aligned to the GRCh37 reference (refdata-b37-2.1.0) using Long Ranger v2.2 and were used to identify single nucleotide polymorphisms (SNPs), phase heterozygous variants, and assign aligned sequencing reads to their haplotype of origin.

### Schizophrenia – NDA collection 2963

Frozen pulverized brain tissues isolated from DLPFC and HIPPO of 21 individuals (11 SCZ and 10 age-matched control) were obtained from the LIBD. Note that although these subjects are shared with collections 2967 and 2966, the majority of these specimens underwent different sequencing workups from the related NDA repositories. The metadata of the shared individuals will be found in the related collections. Samples from six other subjects (five neurotypical and one with unknown condition) not shared with the other collections were also obtained, and underwent single cell sequencing. Nuclei isolation medium was added to tissue samples, which were then homogenized with a dounce homogenizer on ice. Purified nuclei were then incubated with PBS containing 5% BSA and 5 μg/mL Alexa Fluor 488 conjugated anti-NeuN antibody (Millipore Sigma) at 4 °C for 1 h. Nuclei were then stained for 10 μg/mL DAPI and sorted by FACS. Single nuclei from the NeuN− and DAPI-positive population were sorted into 384-well plates containing 1.5 μL of lysis buffer (0.2 M KOH, 0.05 M DTT), alongside two water controls.

Single nuclei underwent whole genome amplification using the PicoPLEX WGA kit, where they were incubated on ice for 10 min, incubated at 65 °C for 10 min, and then cooled to 4 °C, after which 9 μl of sample buffer, 9 μl of reaction buffer, and 1 μl phi29 enzyme (GenomiPhi HY, GE Healthcare) were added to each well. Reactions were incubated at 30 °C for 8 h and then inactivated at 65 °C for 10 min. MDA products were examined for sufficient amplification using a subset of the 47 single copy loci used in Hosono *et al*. 2003 as quality control markers, as they are distributed throughout the genome^41^. MDA samples that passed quality control metrics were subjected to somatic LINE-1-associated variant sequencing (SLAV-seq) library preparation^13^.

As described in Erwin *et al*. 2016, for preparation of SLAV-seq libraries, 10 μg of MDA product was sheared to approximately 500 bp. The sheared DNA was concentrated with Ampure beads (Beckman Coulter) before undergoing capture, annealing and extension with platinum Pfx DNA polymerase (Invitrogen; 94 °C 5 min, 61.5 °C for 30 s, 68 °C for 3 min) and sets of primers relevant to each step. Capture of LINE-1 sequences proceeded using the 5′ biotinylated oligo of sequence ATATACCTAATGCTAGATGACAC*A, with phosphorothioate linkages at the asterisk mark. For annealing, oligos included 5′-AATGATACGGCGACCACCGAGATCTACACNNNNNNNNACACTCTTTCCCTACACGACGCTCTTCCGATC*T and /5Phos/GATCGGAAGAGCGTCGTGTAGGGAAAGAGTGT/3AmM/. PCR amplification (16 cycles) included the PCR primers 5′-CAAGCAGAAGACGGCATACGAGANNNNNNNGTGACTGGAGTTCAGACGTGTGCTCTTCCGATCTNTAACTAACCTGCACAATGTGCAC and 5′-AATGATACGGCGACCACCGAGATCTACAC. Water control MDA products were included in the SLAV-seq procedure as negative controls. The preamplified material was handled in a separate single cell room in a laminar flow hood, and materials used in preamplification, excluding enzymes, sample and reaction buffers, were UV sterilized before use. After purification with Ampure beads, quantification with picogreen and qPCR, then adding 10–20% phiX, the samples underwent paired-end sequencing with either an Illumina HiSeq 2500 or Illumina NovaSeq platform at the Salk next-generation sequencing core^13^.

### Schizophrenia – NDA collection 2965

NeuN+ nuclei isolation was performed on DLPFC tissue samples using fluorescence-activated nuclei sorting. The Illumina PCR-free TruSeq DNA Library Prep kit was used according to the manufacturer’s protocol to produce the DNA libraries of the sorted samples. Constructed libraries were quantified using the KAPA Library Quantification Kit with real-time PCR and were then sequenced on the Illumina HiSeq X Ten platform to yield 150 bp paired-end reads for all samples. Whole genome sequencing of these samples aimed for 200X coverage per sample (Supp. Fig. 1).

Illumina short sequencing reads were processed by a computational pipeline described in detail in Wang *et al*., 2021. Briefly, for each sample, the reads were aligned to the reference sequence GRCh37d5 using bwa (v3.7.16a)^15–17^. Duplicate reads were marked with PICARD (v2.12.1). Indel realignment and base quality score recalibration using GATK (v3.7-0) gave rise to the final BAM file for each sample^18^.

From the final BAM files, single nucleotide variants and small indels were called using GATK (v3.7-0) HaplotypeCaller with–ploidy option 2, 12, or 50 resulting in three raw call sets per sample^19^. The raw call sets were filtered in the following sequential steps: (1) only PASS calls were retained; (2) known germline variants were excluded based on the 1000 Genomes, ExAC, GnomAD, ESP5600, and Kaviar databases^21,22,42,43^; (3) calls located in problematic genomic regions defined as non-P bases of the 1000 Genomes were removed; (4) a binomial test was used to remove likely heterozygous variants: for each variant the p and N parameter of the binomial null distribution was set to 0.5 and the total read count (read depth) at the variant position, respectively, and we removed the variant supporting the null hypothesis at α = 0.00001; (5) a similar binomial hypothesis test was used to filter variant calls at sites with strand bias; (6) similarly, Fisher’s exact test was used to filter out calls with imbalance of strand ratios between reference (REF) and alternative (ALT) bases; (7) multi-allelic calls were removed; and (8) calls at sites with >2.5 copy number, estimated using CNVnator, were also removed^44^.

The implementation of the pipeline used for the present work was designed to run on AWS EC-2 compute instances managed by the AWS ParallelCluster software. The source code is available at https://github.com/bsmn/bsmn-pipeline.

### Schizophrenia – NDA collection 2964

The BP cohort from SMRI included samples from individuals diagnosed with SCZ (n = 35). Details are provided in the previously described BP section.

### Tourette syndrome – NDA collection 2961

The analyzed samples and generated data were de-identified and derived from individuals postmortem.

Brains with Tourette Syndrome sequenced by Yale University were obtained from the Harvard’s Brain and Tissue Resource Center (HBTRC). Normal brains sequenced by Yale University were obtained from NIH NeuroBioBank. The work with those brains was handled in multiple institutes under multiple IRBs. These included the University of Miami Brain Endowment Bank (IRB no. 19920358 (CR0001775)), the University of Maryland Brain and Tissue Bank (IRB no. HM-HP-00042077 and IRB no. 5–58), the Harvard Brain Tissue Resource Center (IRB no. 2015P002028), The Human Brain and Spinal Fluid Resource Center (IRB identification via PCC# 2015-060672 and VA Project# 0002), the Mount Sinai Brain Bank (IRB no. HAR-13-059), and the Brain Tissue Donation Program at the University of Pittsburgh (IRB no. REN14120157/IRB 981146).

For normal brains obtained through autopsy authorization of Yale University, consent to utilize tissues for research and consent to publish was provided by the patient’s next-of-kin according to Connecticut state law and approved protocols of the Yale University BioBank. The research study was approved by the Yale Alzheimer Disease Research Center (ADRC) and was reviewed and deemed exempt by the Yale University Institutional Review Board. The BioBank protocols are in accordance with the ethical standards of Yale University.

Handling of brains originating from and sequenced by the Lieber Institute was conducted under the approved WCG IRB protocol 20111080 titled “Collection of Postmortem Human Brain, Blood and Scalp Samples for Neuropsychiatric Research”.

Data for brains sequenced by Harvard University were publicly available from NIMH Data Archive.

A mass of 1.5 g of postmortem tissue (CTX BA6 or caudate nucleus and putamen) was dissected into 2 mm^2^ cubes per sample to ultimately process NeuN-sorted nuclei fractions using whole genome sequencing. The tissue was lysed and nuclei were extracted following the protocol described by Matevossian, A. & Akbarian, S. 2008^45^. Nuclei were resuspended at 0 °C in PBS and BSA (dilution 1:100). Nuclei isolated from the caudate nucleus and putamen were incubated with the following combination of antibodies: anti-NeuN conjugated to 488 fluorophore (Millipore clone A60, MAB377X; 1:500 dilution) to sort for NeuN+ neurons versus NeuN- glia, and anti-CTIP2 conjugated to 647 fluorophore (Abcam ab18465, 1:250 dilution; conjugation in-house with Alexa Fluor 647 Antibody Labeling Kit, Thermo Fisher, A20186) to separate medium spiny neurons (NeuN+/CTIP2+) from interneurons (NeuN+/CTIP2−). Additionally, these nuclei were incubated with anti-Sox10 antibody (Cell Signaling, 1:500 dilution) and secondary antibody Cy™3 AffiniPure Donkey anti-Rabbit IgG (H + L) (Jackson Laboratory, 711-165-153; 1:200 dilution) to enrich for NeuN-Sox10+ oligodendrocytes and NeuN-Sox10-astrocytes. To isolate cortical interneurons, since cortical interneurons express Sox6, anti-CTIP2 was replaced with anti-Sox6 conjugated to 647 fluorophore antibody (Abcam ab30455, 1:500 dilution; conjugation in-house with Alexa Fluor 647 Antibody Labeling Kit, Thermo Fisher, A20186). Therefore, for the staining of cortical nuclei, anti-Sox6 was added to the previously described anti-NeuN and anti-Sox10 antibodies. Sorting was performed for NeuN+ Sox6+ interneurons, NeuN+ Sox6− pyramidal neurons, NeuN−Sox10+ oligodendrocytes, and NeuN-Sox10-astrocytes.

Nuclei were sorted as fractions into lysis buffer from the DNeasy Blood and Tissue Kit (Qiagen) by FANS (BD FACSAria™ II). DNA was extracted using the DNeasy Blood and Tissue Kit, which included an RNase A treatment step according to the manufacturer’s recommendations. DNA was eluted in 100 µl of elution buffer and the DNA concentration was measured by Qubit (Thermo Fisher).

Library preparation (240 fractions: TruSeq PCR-free 450 bp, 500 ng DNA input, 32 fractions: TruSeq Nano, 450 bp, 200 ng DNA input) and WGS was performed by the New York Genome Center (NYGC) and sequenced using the Illumina HiSeq X platform to produce 150 bp paired-end reads at about 30X coverage (Supp. Fig. 1).

Bulk samples of control and TS-affected brain tissue also underwent whole genome sequencing. A mass of 0.1 g of bulk tissue was incubated at 56 °C overnight in lysis buffer from the DNeasy Blood and Tissue kit and DNA was extracted according to the manufacturer’s recommendations, which included RNase A treatment. DNA was suspended in 200 µl of elution buffer and the DNA concentration was measured by Qubit (Thermo Fisher). Library preparation and WGS were performed by either Macrogen or NYGC. Both sequencing services used Illumina TruSeq DNA PCR-free library kits resulting in paired-end 150 bp reads sequenced to 100X coverage (Supp. Fig. 1). The Macrogen site used 1 µg DNA input, and NYGC used 800 ng of input. Both used Illumina platforms, and NYGC used an Illumina HiSeq X sequencer.

## Data Records

The ten BSMN data repositories are accessible in the NIMH Data Archive (NDA). Collection details are listed in Table 4. Additional metadata and a copy of these access links can also be accessed through the Synapse portal at https://bsmn.synapse.org/Explore/Data. Sample details can be found in Supplementary Tables 1–3.

**Table 4:** The 10 BSMN NDA repositories are listed.

Condition	NDA ID	Collection Name	URL	Num. of Donors	Data Size (TB)
Neurotypical reference brain	2458	Brain Somatic Mosaicism Network Reference Tissue Project	https://nda.nih.gov/edit_collection.html?id=2458	13	24.8
Autism Spectrum Disorder	2962	1/2 Somatic mosaicism and autism spectrum disorder	https://nda.nih.gov/edit_collection.html?id=2962	79	102.6
Autism Spectrum Disorder	2960	2/2 Somatic mosaicism and autism spectrum disorder	https://nda.nih.gov/edit_collection.html?id=2960	40	1.31
Bipolar Disorder; Schizophrenia	2964	Role of somatic mosaicism in autism, schizophrenia, and bipolar disorder brain	https://nda.nih.gov/edit_collection.html?id=2964	597	20.8
Focal Cortical Dysplasia; Neurotypical reference	2968	Mosaicism in focal cortical dysplasias spectrum seen in neuropsychiatric disease	https://nda.nih.gov/edit_collection.html?id=2968	107	57.2
Schizophrenia	2967	1/3 Schizophrenia Genetics and Brain Somatic Mosaicism	https://nda.nih.gov/edit_collection.html?id=2967	108	33.7
Schizophrenia	2966	2/3 Schizophrenia Genetics and Brain Somatic Mosaicism	https://nda.nih.gov/edit_collection.html?id=2966	21	23.6
Schizophrenia	2963	3/3 Schizophrenia Genetics and Brain Somatic Mosaicism	https://nda.nih.gov/edit_collection.html?id=2963	27	0.98
Schizophrenia	2965	Somatic Mosaicism in Schizophrenia and Control Brains	https://nda.nih.gov/edit_collection.html?id=2965	95	67.8
Tourette Syndrome	2961	Somatic Mosaicism in the Brain of Tourette Syndrome	https://nda.nih.gov/edit_collection.html?id=2961	43	69.6

The table includes the collection ID, name, URL, number of donors, approximate repository size, and relevant condition under study for each collection. Note that some donors are shared between some repositories, and some subject IDs may represent experiments, not individuals. For example, collection 2458 lists 13 donors, but some subjects are mixed-DNA experiments, as described in the methods section. Collections 2967, 2966, and 2963 overlap with some shared subjects that underwent different types of sequencing. 1087 unique subject IDs are listed across all BSMN collections.

After obtaining NDA clearance, the data of these collections can be accessed through the primary NDA website as packages, which contain both metadata tables of each collection and the locations of the data files in Amazon Web Services S3 Object Storage. Instructions for obtaining clearance and procuring the data can be found at https://nda.nih.gov/nda/webinars-and-tutorials.html. The Author Contributions section describes PIs and institutions associated with each NDA collection.

Once a data package is downloaded, the set of four key metadata tables per collection can also be reviewed. These files are described in Table 5 and connect subject Globally Unique Identifiers (GUIDs) with relevant de-identified donor, tissue sample, sequencing assay, and related information. These files also contain the S3 links of a variety of data, including WGS, WES, RNA-seq, linked read, single cell WGS-seq, and SLAV-seq of bulk tissue or fractionated samples. Assays used for each condition, along with the number of donors and NDA collection IDs, are shown in Table 6. Note that the use of these assays is not universal for all conditions, donors, and tissue samples, and the metadata tables provide information needed to determine what S3 links are most relevant. The formatting of these tables is standardized with NDA policy.

**Table 5:** The list of metadata tables available for each NDA collection after deploying a data package for AWS Oracle access.

Table Name	Description
GENOMICS_SUBJECT02	Connects samples with GUIDs, relevant donor information, and sample types.
GENOMICS_SAMPLE03	Associates GUID, sample, experiment IDs, data files, and AWS S3 links together.
NICHD_BRB02	Contains GUIDs and tissue sample information.
OMICS_EXPERIMENT	Describes experiment IDs, assays, and sequencing platform information.

**Table 6:** This table describes the number of donors, collections, and assays associated with each condition.

Assays include whole genome (WGS), whole exome (WES), RNA-seq (RNA), single cell (SC), linked read (LR), and customized (CUS) sequencing methods. Custom sequencing includes SLAV-seq of C2963. Two neurotypical, thoroughly sequenced subjects that can be used as references are present in collections 2458 and 2698. Neurotypical controls are marked as “No Condition, Not Reference.” The diagnostic category Other contains n = 4 Unknown phenotype (one in C2963, three in C2964), n = 8 major depressive disorder, n = 25 recurrent unipolar depression (RUDD), n = 30 other mental health diagnosis that is not BP, but not otherwise specified, and n = 1 CADASIL for collection 2964.

File and data types are described in the GENOMICS_SAMPLE03 metadata file of each NDA collection. Files within the repository include sequence data (FASTQs), alignment files (BAM/CRAM), the index files (BAI/CRAI/TBI), alignment summary text files, and files for variant calls (VCFs). The availability of these file types is not universal across repositories, however. See Supp. Table 3 for available file types per sample within each collection.

In addition to NDA collections, which are repositories specific to an entire project, cross-collection subsets of data can be established using the NDA’s Study feature. NDA Studies 967 and 814 will be particularly useful to researchers interested in the BSMN data. Study 967 contains all data of the BSMN^46^. Study 814 contains data that was uniformly processed using an identical alignment pipeline across the entire consortium^47^. This workflow starts with separating fastq files by flow cell lane, with the individual lanes then being aligned to GRCh37d5 with bwa v3.7.16a to obtain read group identifiers by lane. The alignment files were sorted by read group then merged by sambamba v0.6.7 before PICARD v2.12.1 was used to mark PCR duplicates. Following GATK best practices (v3.7), indel realignment and base quality recalibration were then performed to yield the final alignment files. The BSMN Pipeline applied to the Study 814 data is available in Github Repository https://github.com/bsmn/bsmn-pipeline (Table 7)^7,8^.

**Table 7:** Computational tools developed by the BSMN Collaborators that are publicly available for community use.

Code Availability - Repositories and Tools		
Computational Tool	Repository	Description
BSMN Pipeline^8^	https://github.com/bsmn/bsmn-pipeline	A pipeline for aligning and calling variants for the BSMN data.
CNVpytor^50^	https://github.com/abyzovlab/CNVpytor	A tool for copy number alteration and allelic imbalance analyses.
MosaicForecast^9^	https://github.com/parklab/MosaicForecast/	A machine-learning pipeline for finding mosaic SNVs without the need for a control via phasing and random forest modeling.
Panel of Normals mask^8^	10.5281/zenodo.4321679	A panel of normals filter mask for detecting false positives mosaic SNVs. Generated using 30X WGS data from the 10000 Genomes Project.
Phase Mosaic^10^	https://bitbucket.org/donald_freed/phase-mosaic/src/master/	A tool to determine if de novo variants are mosaic using phasing of local heterogeneous haplotypes.
Find Denovo^10^	https://github.com/pevs/find_denovo	A tool for parsing putative de novo variants from a VCF or BCF file.
RetroSom^11^	https://github.com/XiaoweiZhuJJ/RetroSom	A machine-learning tool for detecting LINE-1 insertions in high depth WGS data.
DeepMosaic^12^	https://github.com/Virginiaxu/DeepMosaic	A machine-learning tool for visualizing and predicting SNV mosaicism without controls.
PySlavSeq^13^.	https://github.com/apuapaquola/pyslavseq	A tool for working with SLAV-seq data.
Longboard^13^	https://github.com/PatrickJReed/Longboard	A machine-learning pipeline for detecting LINE-1 insertions in SLAV-seq data.

## Technical Validation

**Fig. 1:**
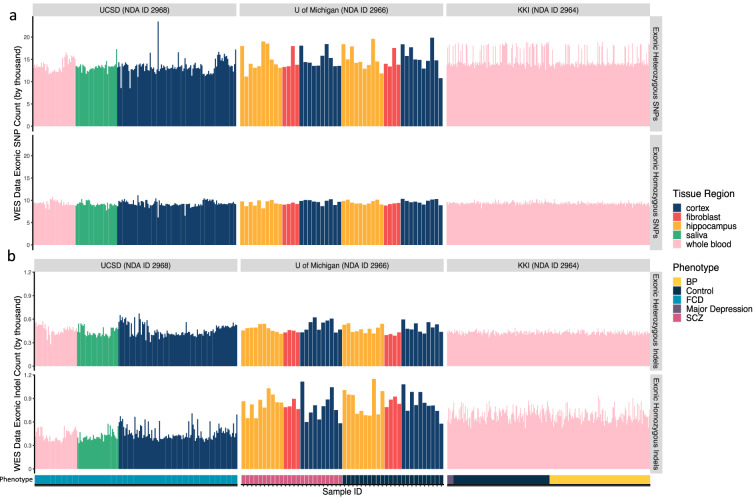
Heterozygous and homozygous (**a**) SNP and (**b**) indel counts in targeted exonic regions. Data represents uniformly processed WES data of cohorts from the University of San Diego (NDA ID 2968), University of Michigan (NDA ID 2966), and Kennedy Krieger Institute (KKI; NDA ID 2964). Phenotype for each cohort is marked at the bottom of the figure in a rugchart.

**Fig. 2:**
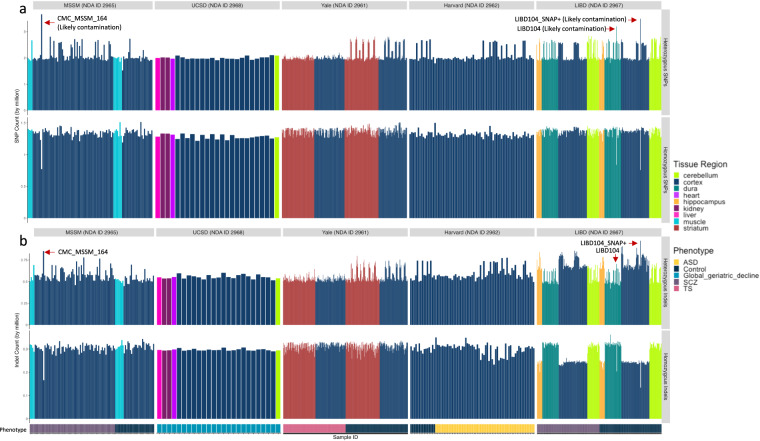
Heterozygous and homozygous (**a**) SNP and (**b**) indel counts in the uniformly processed WGS data in the cohorts from Mount Sinai School of Medicine (MSSM; NDA ID 2965), University of San Diego (UCSD; NDA ID 2968), Yale University (NDA ID 2961), Harvard University (NDA ID 2962), and the Lieber Institute for Brain Development (LIBD; NDA ID 2967). Phenotype is marked at the bottom in a rugchart. Some samples with possible contamination are labeled.

Data quality and purity were assessed in a few different ways. Germline variants were called for every WES and WGS sample that underwent uniform processing (Supp. Tables 1, 2), which were used to determine ethnic background and quality of the data. (Note that data that was not included in the uniform processing pipeline, such as the NRGR and SMRI datasets of collection 2964 or the SLAV-seq data targeting LINE-1s of 2963, are still available through the repository ID but are not represented in the following described figures. See Supp. Table 3 for all sample IDs, regardless of uniform processing status.) The counts of these calls, which included heterozygous and homozygous SNPs and indels, were compared across the data, and were found to be relatively consistent (Figs. 1, 2). Overall, two groups were observed with higher and lower counts, with consistent germline variation matching expectations for populations of African and Caucasian/Asian descent^22^. More consistency was observed for SNPs, likely reflected in more bias in capturing and calling indels, which has been observed by other studies^21^.

**Fig. 3:**
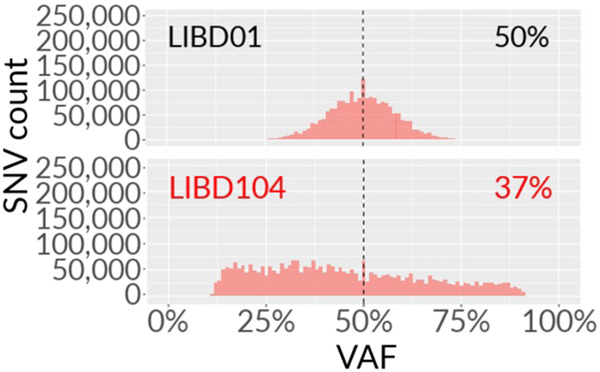
Variant allele frequencies (VAFs) for heterozygous SNPs were obtained for each sample using GATK HaplotypeCaller with ploidy 2. Two example distributions are shown here for samples LIBD01 and LIBD104 (collection 2967). The distribution is shifted from 50% and does not appear normally distributed for LIBD104 suggesting contamination of the sequenced sample with unrelated DNA.

Some outliers of SNP counts prompted further investigation, and are marked in Fig. 2. These included samples CMC_MSSM_164 from the Mount Sinai School of Medicine (MSSM; NDA ID 2965), and Br5459 of LIBD (NDA ID 2967). Variant allele frequency (VAF) distributions were collected for the WGS data (Fig. 3; Supp. Fig. 3). Heterozygous SNP output from GATK HaplotypeCaller with ploidy 2 were plotted, with the expectation of the peak distribution to sit at a VAF of 50%. Deviation may hint at contamination from an alternative source, shown with two examples in Fig. 3, where one of the two distributions indicates a deviation. The outliers of the SNP and indel counts marked in Fig. 2 show similar deviations. We marked other potentially contaminated WGS samples with a peak of VAF distribution deviating from 50% by more than 2% in Supp. Figure 3 with red sample titles. Mapping quality and percentages are listed in Supp. Tables 1, 2. If the samples were potentially contaminated (via the VAF distribution deviation), they were also marked in Supp. Table 1.

**Fig. 4:**
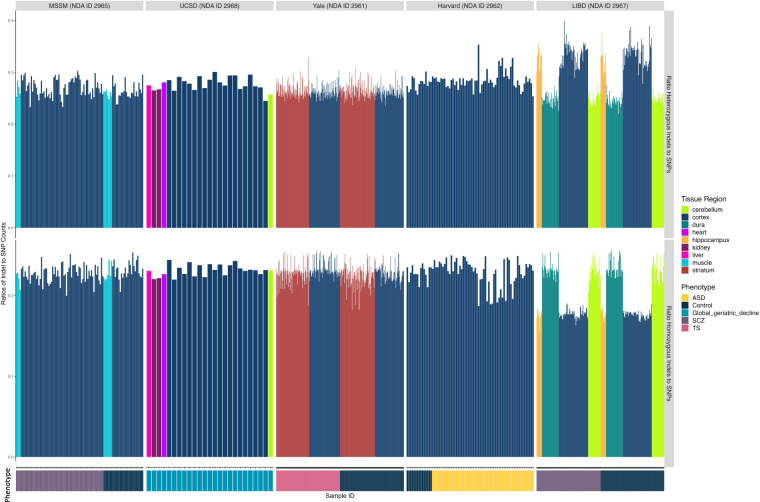
Ratios of heterozygous and homozygous indels to SNPs across all cohorts for all uniformly processed WGS data.

To check for variation in data quality, we also considered the fraction of indels to SNPs. The fraction of indels seen were consistent with recent studies^21^. The ratios of heterozygous and homozygous indel to SNP counts per sample are shown in Fig. 4. Overall, these ratios were consistent within the datasets. Variations were likely reflecting some variation in DNA extraction, library preparation, or sequencing. However, the differences were not affiliated with evidence of contamination.

**Fig. 5:**
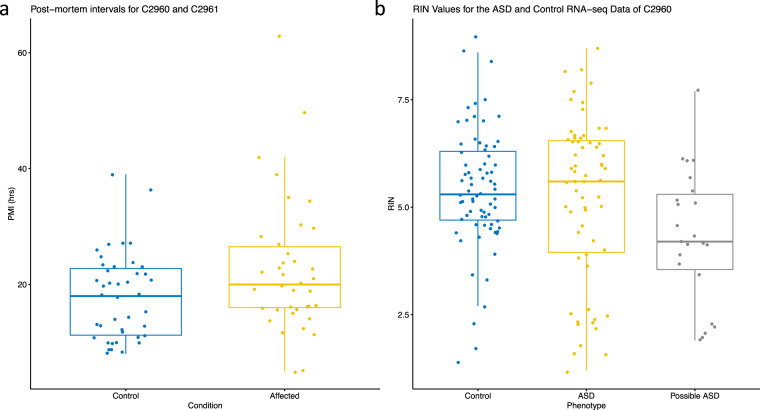
Quality of RNA-seq data. (**a**) Postmortem interval (PMI) of affected (n = 38) and control (n = 42) individuals in the two Yale cohorts (collections 2960 and 2961), which were the only cohorts with such a data type. Differences were not statistically significant (p > 0.05). (**b**) RIN values of ASD-affected (n = 63 ASD, n = 23 suspected ASD samples) and control (n = 73 neurotypical control samples) RNA-seq samples from Yale cohort 2960.

Postmortem intervals (PMIs) were also reported for two repositories (2960 and 2961; Fig. 5a). Median PMIs for these datasets were approximately 20hrs. RIN values were also collected for the RNA-seq data of repository 2960 (Fig. 5b). Median RINs were found to be approximately 5.0.

In studies where somatic mosaic variants were reported, validation was typically performed using targeted amplicon sequencing. PCR primers were designed in silico in the range of 200 to 500 bp outside of the target. PCR-amplified targets were isolated using either agarose gel electrophoresis or a manufactured kit, and the isolated sample was re-sequenced on either a MiSeq platform or an Ion Torrent Personal Genome Machine. Control tissues were used for comparison to validated somatic variant-containing samples. Targets were generally selected based on sample availability, ability to use PCR primers at the desired location, functional significance, and range of loci^5,8^.

Additional methods of somatic mosaic variant validation included targeting multiple somatic calls at once with multiplex PCR target amplification using the CleanPlex Custom NGS Panel (Paragon Genomics) for resequencing on a MiSeq platform^8^. Additionally, digital droplet PCR (ddPCR) was applied to selected calls for validation and variant allele frequencies^8,11^. In cases of somatic mosaic retrotransposon calls, nested PCR and Sanger sequencing of the calls were also applied for further validation of the insertions^11^. Other technical validation information is detailed in Erwin *et al*. and Breuss *et al*.^13,14^.

## Usage Notes

The BSMN data provide a rich source of data for further studies of mosaic variation, including multiple platforms (e.g. WES, WGS), sequencing depths, and genomic DNA sources (Table 8). NDA collection 2458 contains the WGS, WES, linked read, and single cell data of neurotypical reference brain LIBD 5154 for bulk brain tissue, NeuN+/NeuN− fractions, and dural fibroblasts. Additionally, NDA collection 2968 contains somatic mosaicism exploration of multiple samples of a mature neurotypical brain with WGS sequencing of bulk and fractionated tissue, as well as MDA and single cell MDA data of certain fractions and tissue types. The somatic single nucleotide variants were validated by PCR target amplification. The reference brain of 2458 and the mature individual in 2968 could be used either as controls for other studies or as data for the development of sensitive bioinformatics tools.

**Table 8:** Lead investigator (PI) and institution associated with each NDA collection.

PI	Institution	NDA ID
Multiple^a^	Multiple^a^	2458
Christopher A. Walsh	Harvard University	2962
Nenad Sestan	Yale University	2960
Jonathan Pevsner	Kennedy Krieger Institute (KKI)	2964
Joseph G. Gleeson	University of San Diego (UCSD)	2968
Daniel R. Weinberger	Lieber Institute for Brain Development (LIBD)	2967
John V. Moran	University of Michigan (U Mich)	2966
H. Gage	Salk Institute	2963
Andrew Chess	Icahn School of Medicine at Mount Sinai (MSSM)	2965
Flora M. Vaccarino	Yale University	2961

^a^The data of collection 2458 was produced across the entire consortium. PIs of 2458 include Alexej Abyzov, Schahram Akbarian, Tomas Marqués-Bonet, Andrew Chess, Fred H. Gage, Joseph G. Gleeson, Jeffrey M. Kidd, Ryan E. Mills, John V. Moran, Peter J. Park, Mette A. Peters, Jonathan Pevsner, Nenad Sestan, Alexander E. Urban, Flora M. Vaccarino, Christopher A. Walsh, Daniel R. Weinberger.

The BSMN findings and methods can be applied to the study of other neuropsychiatric conditions or any other condition in which mosaicism is implicated such as cancer, or to understand the nature of both somatic mosaicism and germline variation in the genetic diversity of individuals. The data can be used to explore the complex genetic spectrum of these conditions in conjunction with other data as well, such as PsychENCODE and the CommonMind consortia^48,49^.

### Supplementary information

Supplementary figures

Supplementary tables

## Data Availability

Computational tools have been developed for processing BSMN data. A list of these tools and their repositories is available on the Synapse site at https://bsmn.synapse.org/Explore/Tools and is included in Table 7.

## References

[1] Jourdon A, Fasching L, Scuderi S, Abyzov A, Vaccarino FM (2020). The role of somatic mosaicism in brain disease. Curr. Opin. Genet. Dev..

[2] D’Gama AM, Walsh CA (2018). Somatic mosaicism and neurodevelopmental disease. Nat. Neurosci..

[3] McConnell MJ (2017). Intersection of diverse neuronal genomes and neuropsychiatric disease: The Brain Somatic Mosaicism Network. Science.

[4] Rodin RE, Walsh CA (2018). Somatic mutation in pediatric neurological diseases. Pediatr. Neurol..

[5] Rodin RE (2021). The landscape of somatic mutation in cerebral cortex of autistic and neurotypical individuals revealed by ultra-deep whole-genome sequencing. Nat. Neurosci..

[6] Sherman MA (2021). Large mosaic copy number variations confer autism risk. Nat. Neurosci..

[7] Bae T (2022). Analysis of somatic mutations in 131 human brains reveals aging-associated hypermutability. Science.

[8] Wang Y (2021). Comprehensive identification of somatic nucleotide variants in human brain tissue. Genome Biol..

[9] Dou Y (2020). Accurate detection of mosaic variants in sequencing data without matched controls. Nat. Biotechnol..

[10] Freed D, Pevsner J (2016). The contribution of mosaic variants to autism spectrum disorder. PLoS Genet..

[11] Zhu X (2021). Machine learning reveals bilateral distribution of somatic L1 insertions in human neurons and glia. Nature Neurosc.

[12] Yang X (2023). DeepMosaic: control-independent mosaic single nucleotide variant detection using deep convolutional neural networks. Nature Biotechnolo.

[13] Erwin JA (2016). L1-associated genomic regions are deleted in somatic cells of the healthy human brain. Nat. Neurosci..

[14] Breuss MW (2022). Somatic mosaicism in the mature brain reveals clonal cellular distributions during cortical development. Nature.

[15] Li H, Durbin R (2009). Fast and accurate short read alignment with Burrows-Wheeler transform. Bioinformatics.

[16] Li H, Durbin R (2010). Fast and accurate long-read alignment with Burrows-Wheeler transform. Bioinformatics.

[17] Genovese G, Handsaker RE, Li H, Kenny EE, McCarroll SA (2013). Mapping the human reference genome’s missing sequence by three-way admixture in Latino genomes. Am. J. Hum. Genet..

[18] McKenna A (2010). The Genome Analysis Toolkit: a MapReduce framework for analyzing next-generation DNA sequencing data. Genome Res..

[19] Poplin, R. *et al*. Scaling accurate genetic variant discovery to tens of thousands of samples. Preprint at 10.1101/201178 (2018).

[20] Cibulskis K (2013). Sensitive detection of somatic point mutations in impure and heterogeneous cancer samples. Nat. Biotechnol..

[21] Karczewski KJ (2020). The mutational constraint spectrum quantified from variation in 141,456 humans. Nature.

[22] 1000 Genomes Project Consortium (2015). A global reference for human genetic variation. Nature.

[23] Kang HJ (2011). Spatio-temporal transcriptome of the human brain. Nature.

[24] Roberson EDO, Pevsner J (2009). Visualization of shared genomic regions and meiotic recombination in high-density SNP data. PLoS One.

[25] Purcell S (2007). PLINK: a tool set for whole-genome association and population-based linkage analyses. Am. J. Hum. Genet..

[26] Freed, D., Aldana, R., Weber, J. A. & Edwards, J. S. The sentieon genomics tools - a fast and accurate solution to variant calling from next-generation sequence data. Preprint at 10.1101/115717 (2017).

[27] Fromer M (2012). Discovery and statistical genotyping of copy-number variation from whole-exome sequencing depth. Am. J. Hum. Genet..

[28] Talevich E, Shain AH, Botton T, Bastian BC (2016). CNVkit: genome-wide copy number detection and visualization from targeted DNA sequencing. PLoS Comput. Biol..

[29] Benjamin, D. *et al*. Calling somatic SNVs and indels with Mutect2. Preprint at 10.1101/861054 (2019).

[30] Huang AY (2017). MosaicHunter: accurate detection of postzygotic single-nucleotide mosaicism through next-generation sequencing of unpaired, trio, and paired samples. Nucleic Acids Res..

[31] Eberle MA (2017). A reference data set of 5.4 million phased human variants validated by genetic inheritance from sequencing a three-generation 17-member pedigree. Genome Res..

[32] Darby CA (2019). Samovar: single-sample mosaic single-nucleotide variant calling with linked reads. iScience.

[33] Blümcke I (2011). The clinicopathologic spectrum of focal cortical dysplasias: A consensus classification proposed by an ad hoc Task Force of the ILAE Diagnostic Methods Commission. Epilepsia.

[34] Tarasov A, Vilella AJ, Cuppen E, Nijman IJ, Prins P (2015). Sambamba: fast processing of NGS alignment formats. Bioinformatics.

[35] Zarate, S. *et al*. Parliament2: Accurate structural variant calling at scale. *Gigascience* **9** (2020). 10.1093/gigascience/giaa145 PMC7751401 33347570

[36] Chiang C (2015). SpeedSeq: ultra-fast personal genome analysis and interpretation. Nat. Methods.

[37] Jeffares DC (2017). Transient structural variations have strong effects on quantitative traits and reproductive isolation in fission yeast. Nat. Commun..

[38] Kim D, Paggi JM, Park C, Bennett C, Salzberg SL (2019). Graph-based genome alignment and genotyping with HISAT2 and HISAT-genotype. Nat. Biotechnol..

[39] Liao Y, Smyth GK, Shi W (2014). featureCounts: an efficient general purpose program for assigning sequence reads to genomic features. Bioinformatics.

[40] Bray NL, Pimentel H, Melsted P, Pachter L (2016). Near-optimal probabilistic RNA-seq quantification. Nat. Biotechnol..

[41] Hosono S (2003). Unbiased whole-genome amplification directly from clinical samples. Genome Res..

[42] Karczewski KJ (2017). The ExAC browser: displaying reference data information from over 60 000 exomes. Nucleic Acids Res..

[43] Glusman G, Caballero J, Mauldin DE, Hood L, Roach JC (2011). Kaviar: an accessible system for testing SNV novelty. Bioinformatics.

[44] Abyzov A, Urban AE, Snyder M, Gerstein M (2011). CNVnator: an approach to discover, genotype, and characterize typical and atypical CNVs from family and population genome sequencing. Genome Res..

[45] Matevossian, A. & Akbarian, S. Neuronal nuclei isolation from human postmortem brain tissue. *J. Vis. Exp*. e914 (2008). 10.3791/914 PMC3233860 19078943

[46] (2023). National Institutes of Health, National Institute of Mental Health (NIMH) Data Archive Repository.

[47] (2021). National Institutes of Health, National Institute of Mental Health (NIMH) Data Archive Repository.

[48] Wang D (2018). Comprehensive functional genomic resource and integrative model for the human brain. Science.

[49] Hoffman GE (2019). CommonMind Consortium provides transcriptomic and epigenomic data for Schizophrenia and Bipolar Disorder. Sci Data.

[50] Suvakov M, Panda A, Diesh C, Holmes I, Abyzov A (2021). CNVpytor: a tool for copy number variation detection and analysis from read depth and allele imbalance in whole-genome sequencing. GigaScience.

