# Supplementary material for: Genomic data resources of the Brain Somatic Mosaicism Network for neuropsychiatric diseases
Source: Sci Data. 2023 Nov 20;10:813. doi: 10.1038/s41597-023-02645-7 (PMC10662356; doi:10.1038/s41597-023-02645-7)

scientific **data**

Supplementary Materials for

Genomic data resources of the Brain Somatic Mosaicism Network for neuropsychiatric diseases

McKinzie A. Garrison *et al.*

Corresponding author: Alexej Abyzov, abyzov.alexej@mayo.edu

Pertaining to data hosted by the NIMH Data Archive:
Study 967 (DOI: 10.15154/1519293) & Study 814 (DOI: 10.15154/1506068)

The PDF file includes:

Supplementary Figures S1 to S3.

Other Supplementary Materials for this manuscript include the following:

Supplementary Tables S1 to S3.

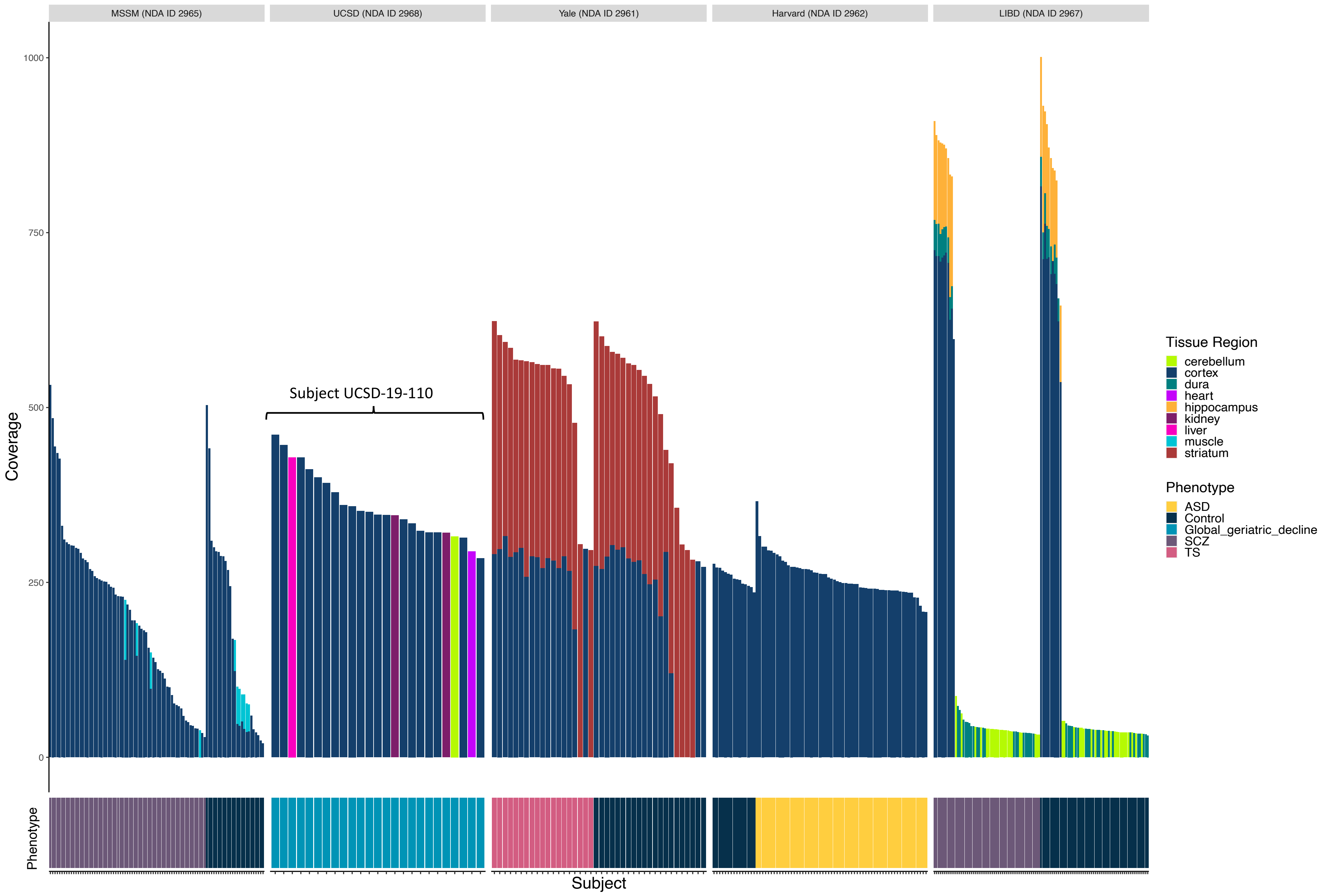

Supplementary Figure 1. Stacked barchart of coverage per subject for each cohort with WGS data, including data for cohorts from the Mount Sinai School of Medicine (MSSM), University of California San Diego (UCSD), Yale University, Harvard University, and the Lieber Institute for Brain Development (LIBD). The UCSD section solely describes samples from a single subject, UCSD-19-110. The barplot is colored for tissue of origin, and phenotype is colored by a rugchart at the bottom for subjects affected by autism spectrum disorder (ASD), global geriatric decline, schizophrenia (SCZ), Tourette syndrome (TS), and control subjects.

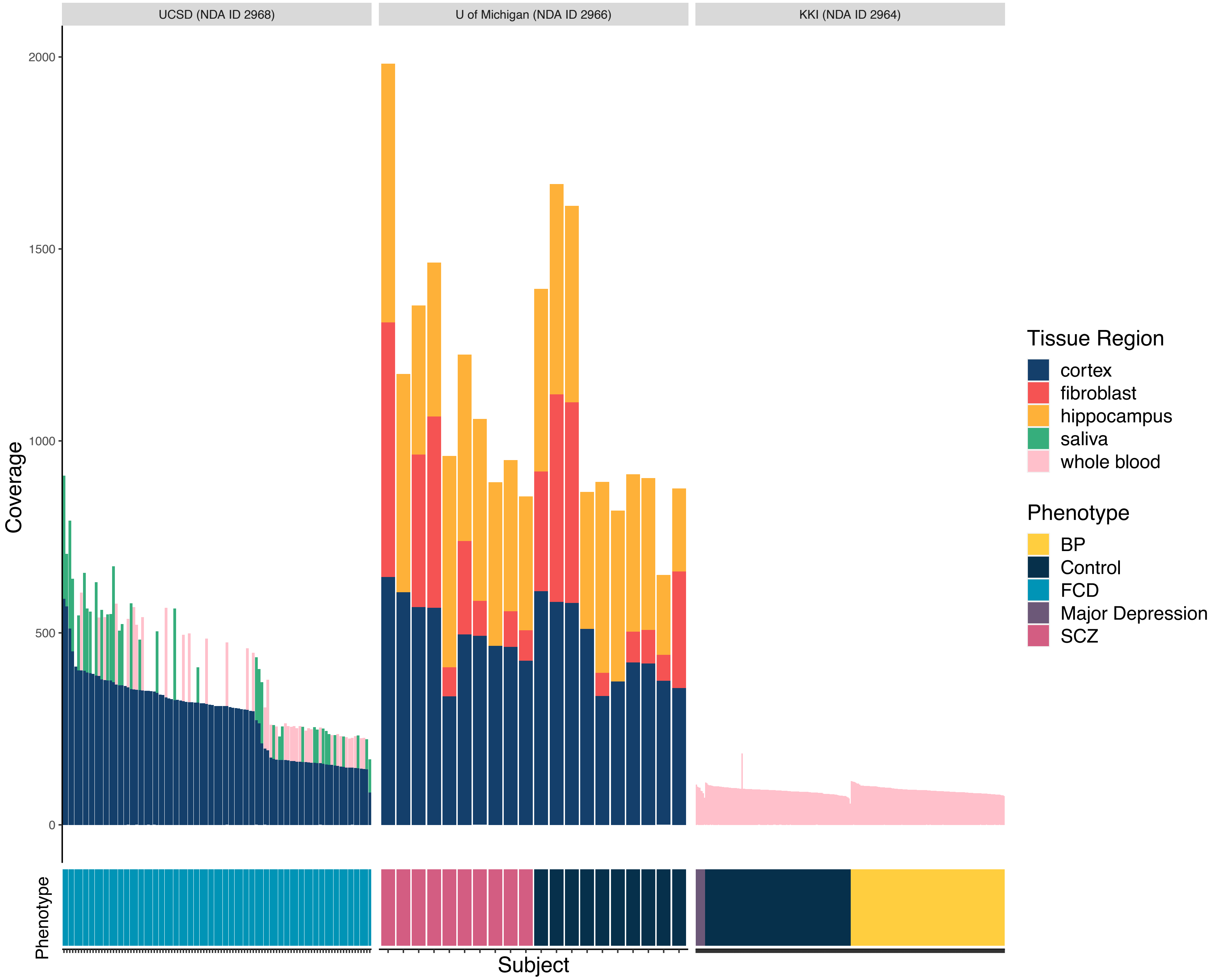

Supplementary Figure 2. Coverage of the uniformly processed WES data for cohorts from University of California San Diego (NDA ID 2968), University of Michigan (NDA ID 2966), and Kennedy Krieger Institute (NDA ID 2964). Phenotype is shown at the bottom of the graph for affected (BP = bipolar disorder, FCD = focal cortical dysplasia, SCZ = schizophrenia, and major depression disorder) and control subjects.

Supplementary Figure 3. Variant allele frequency (VAF) distributions of the heterozygous SNPs of each sample in the Harvard (NDA ID 2962), MSSM (NDA ID 2965), LIBD (NDA ID 2967), Yale (NDA ID 2961), and UCSD (NDA ID 2968) datasets. Most distributions have a median VAF of 50 ± 2%. Samples with a median outside this short range were determined to be likely contaminated. Potentially contaminated samples are marked with red titles.

Supp. Fig 3. (a) Harvard (NDA ID 2962) dataset VAF distributions of heterozygous SNPs.

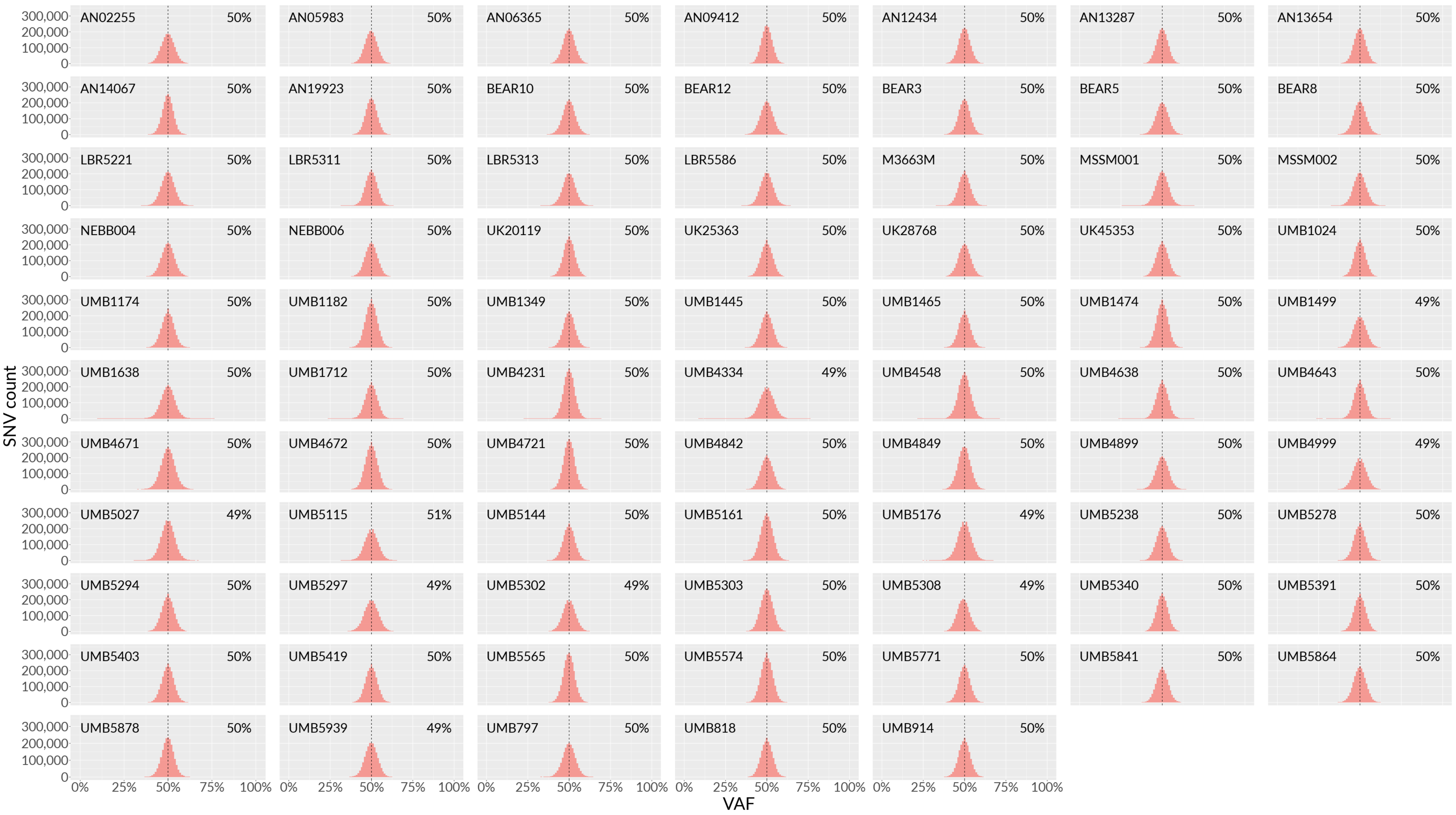

Supp. Fig 3. (b) MSSM (NDA ID 2965) dataset VAF distributions of heterozygous SNPs. Some samples have a bumpy, stepwise appearance. This bumpy appearance is caused by shallow, uneven coverage and lower data quality compared to samples in the same cohort, resulting in variant allele frequencies that are stepwise rather than continuous. These samples still have a median VAF around 50% and are not likely contaminated.

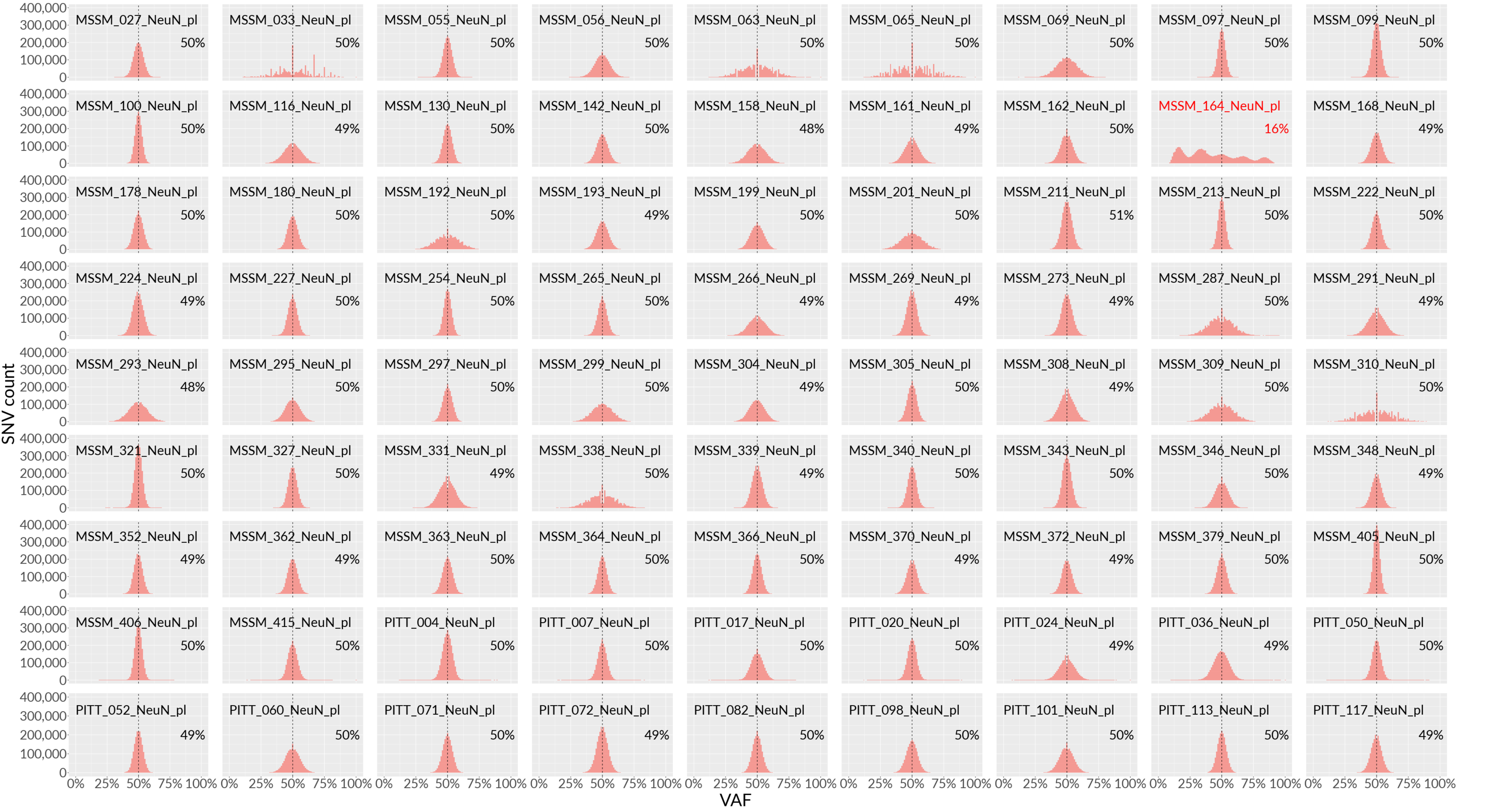

Supp. Fig 3. (b continued) MSSM (NDA ID 2965) dataset VAF distributions of heterozygous SNPs.

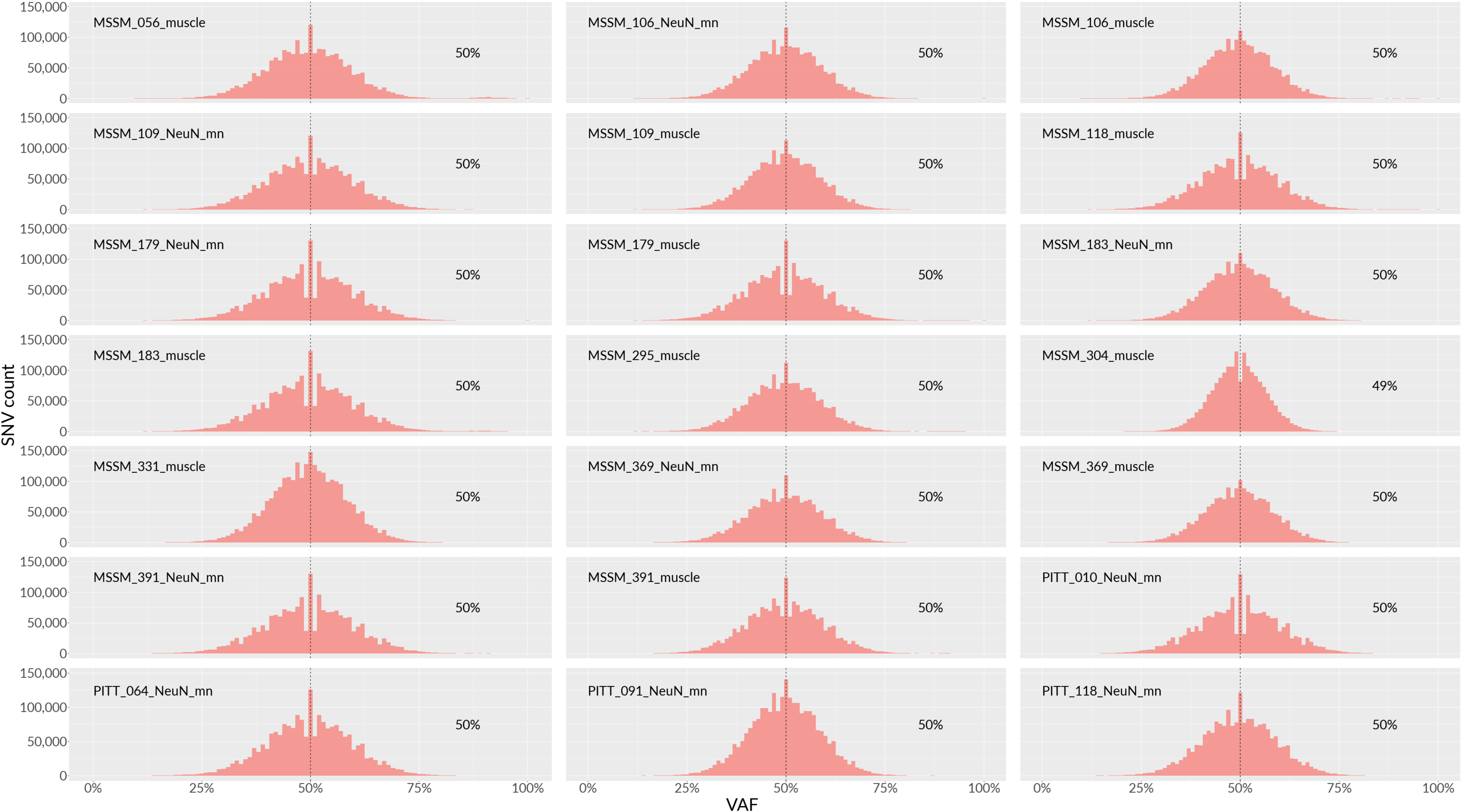

Supp. Fig 3. (c) LIBD (NDA ID 2967) dataset VAF distributions of heterozygous SNPs.

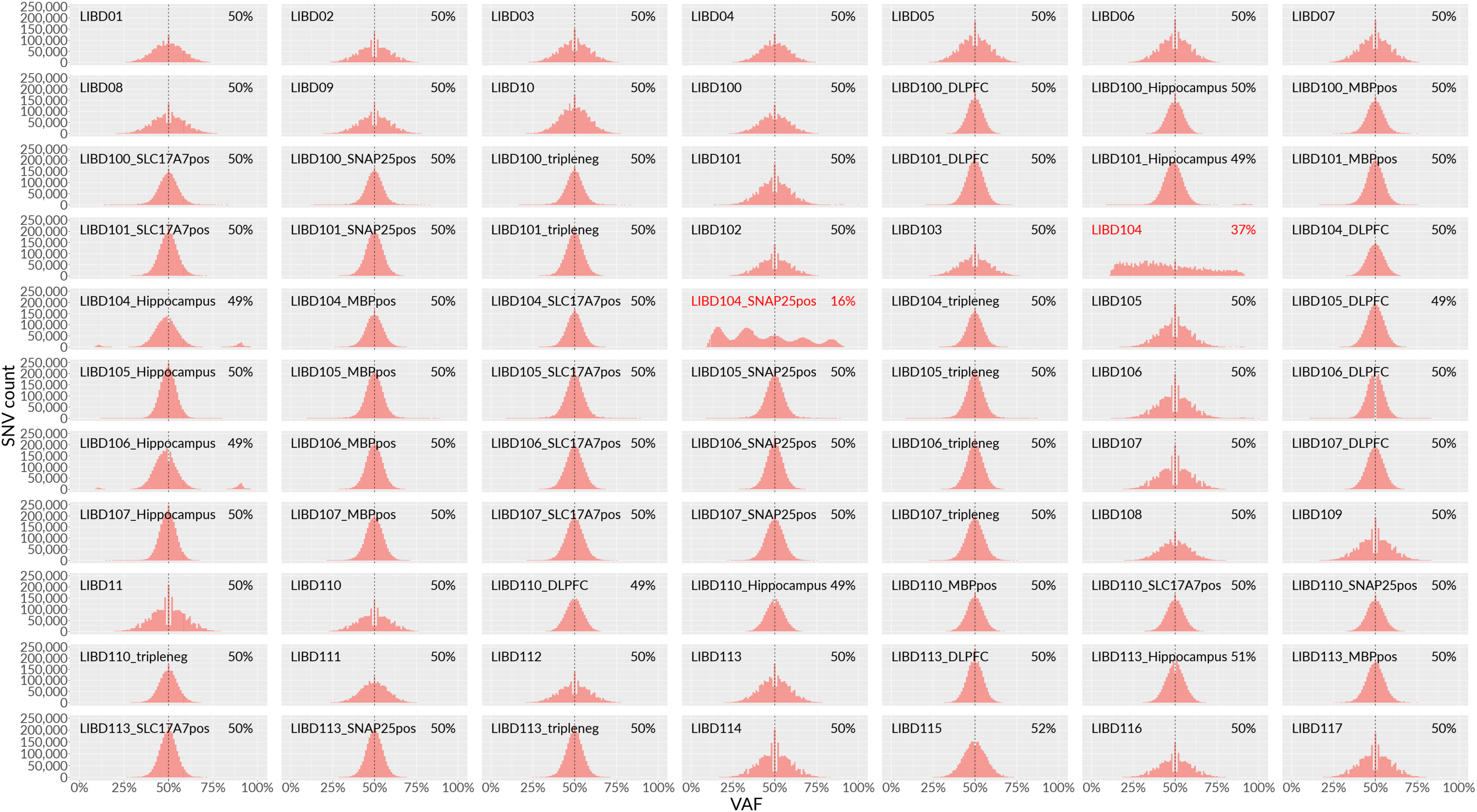

Supp. Fig 3. (c continued) LIBD (NDA ID 2967) dataset VAF distributions of heterozygous SNPs.

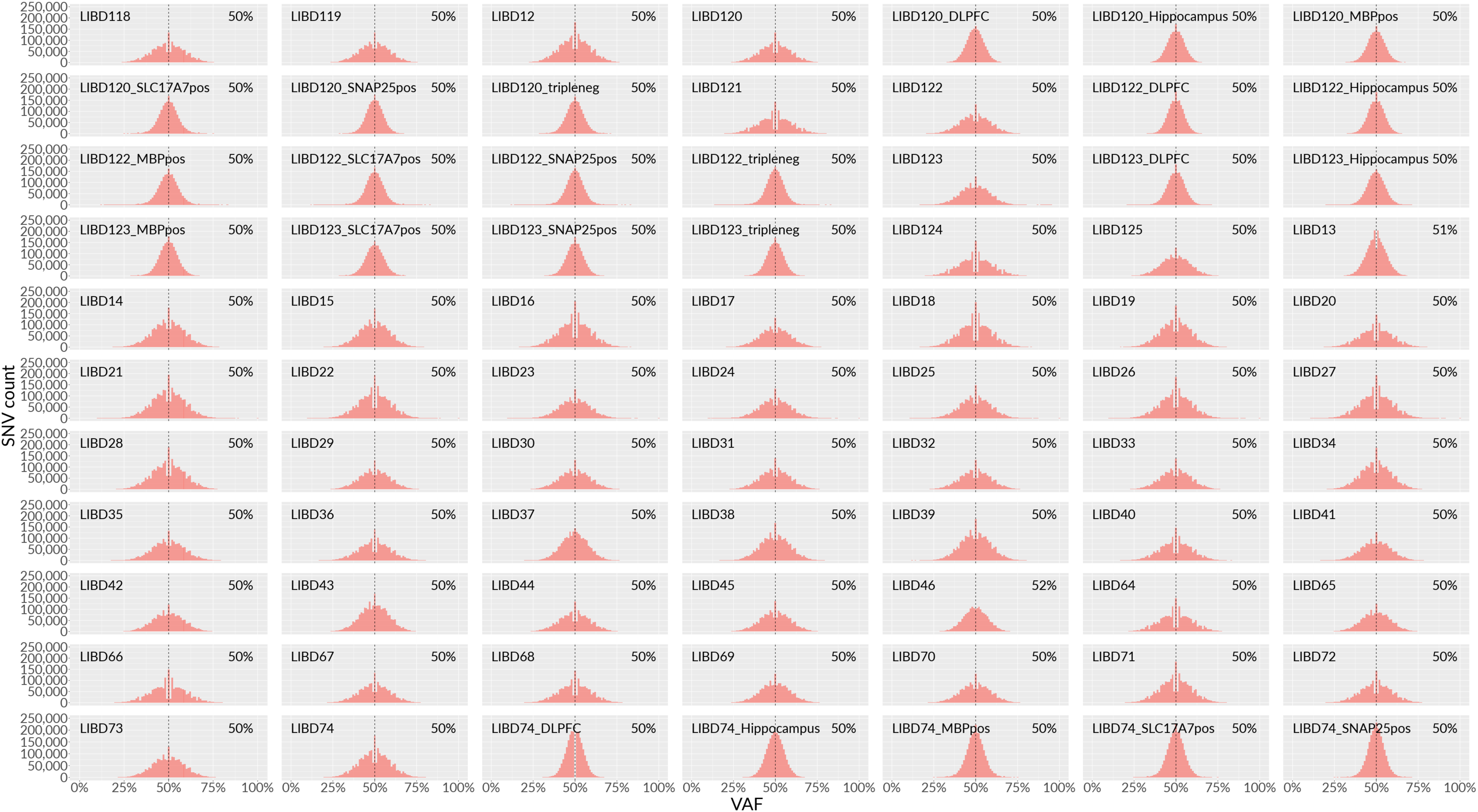

Supp. Fig 3. (c continued) LIBD (NDA ID 2967) dataset VAF distributions of heterozygous SNPs.

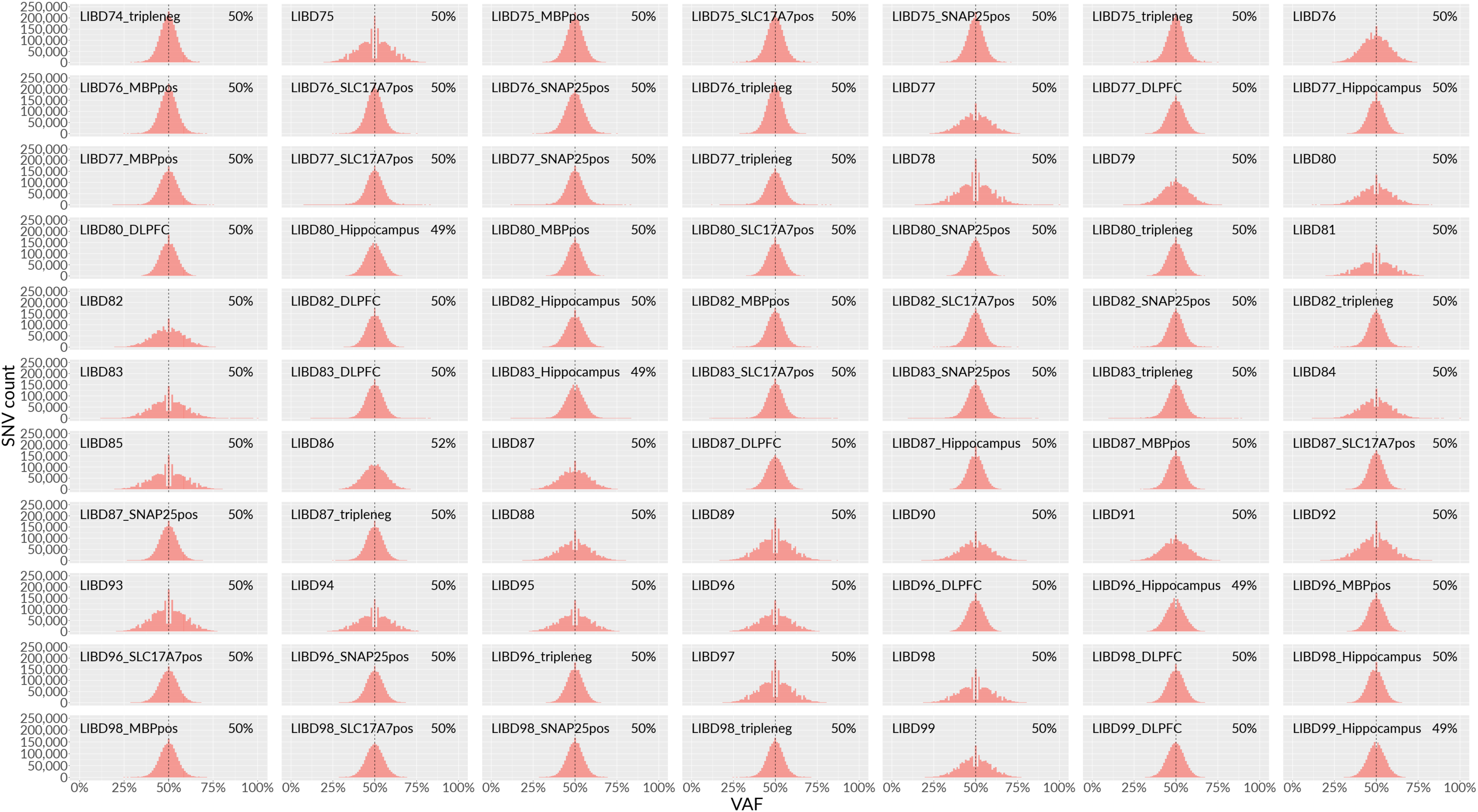

Supp. Fig 3. (c continued) LIBD (NDA ID 2967) dataset VAF distributions of heterozygous SNPs.

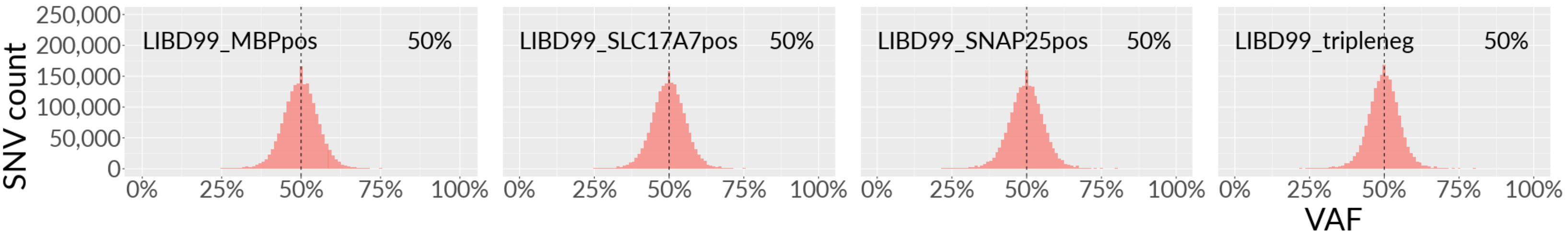

Supp. Fig 3. (d) Yale (NDA ID 2961) dataset VAF distributions of heterozygous SNPs.

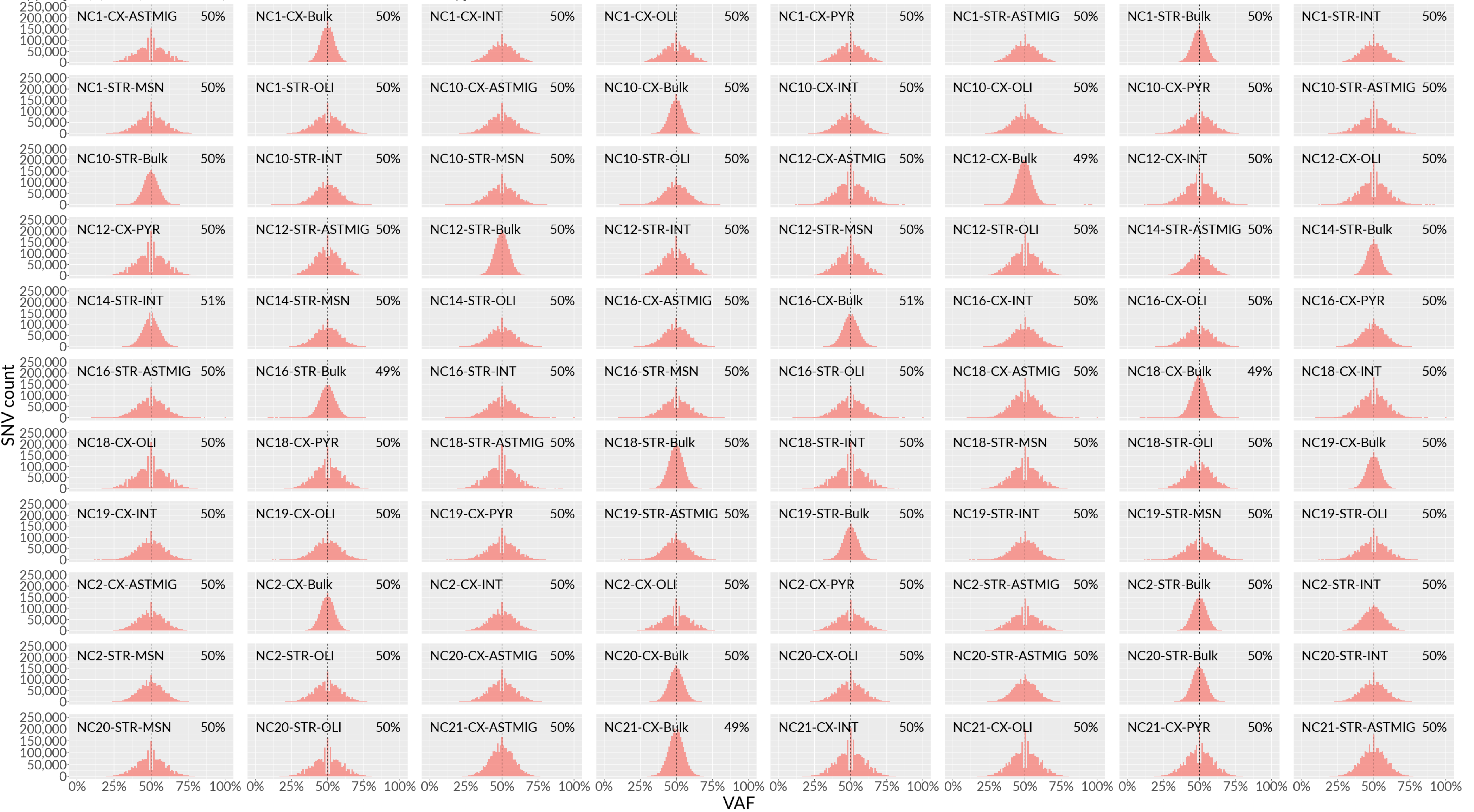

Supp. Fig 3. (d continued) Yale (NDA ID 2961) dataset VAF distributions of heterozygous SNPs.

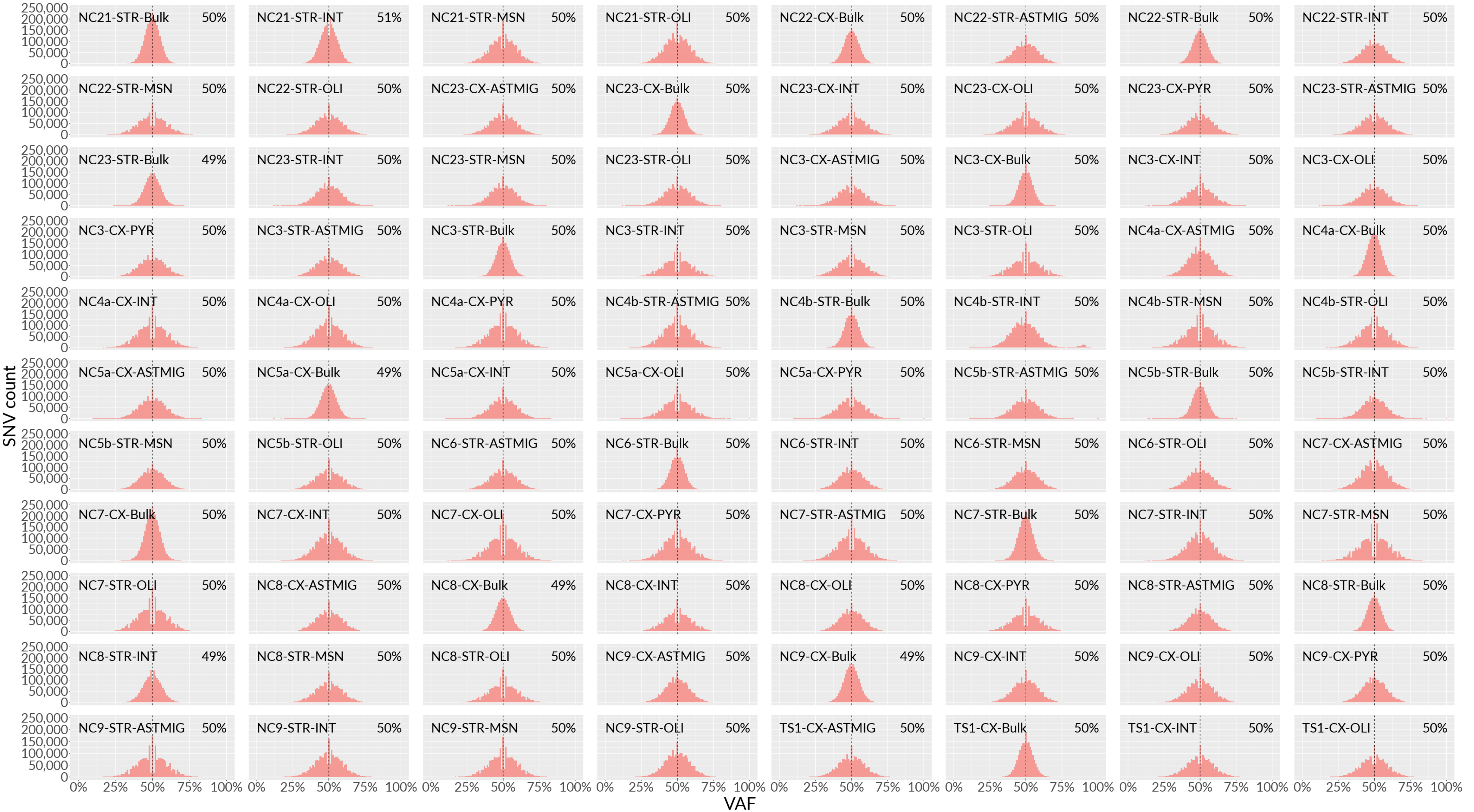

Supp. Fig 3. (d continued) Yale (NDA ID 2961) dataset VAF distributions of heterozygous SNPs.

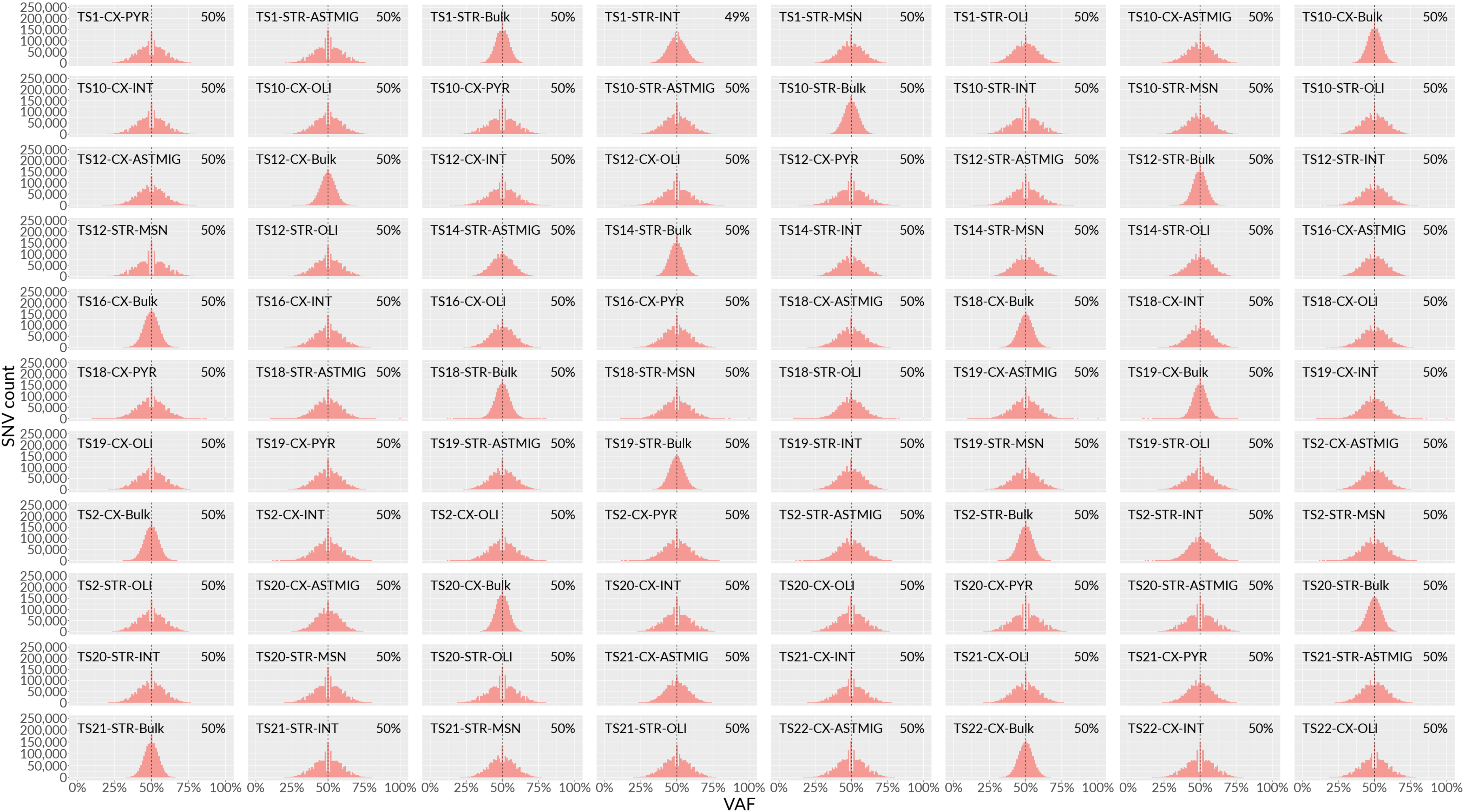

Supp. Fig 3. (d continued) Yale (NDA ID 2961) dataset VAF distributions of heterozygous SNPs.

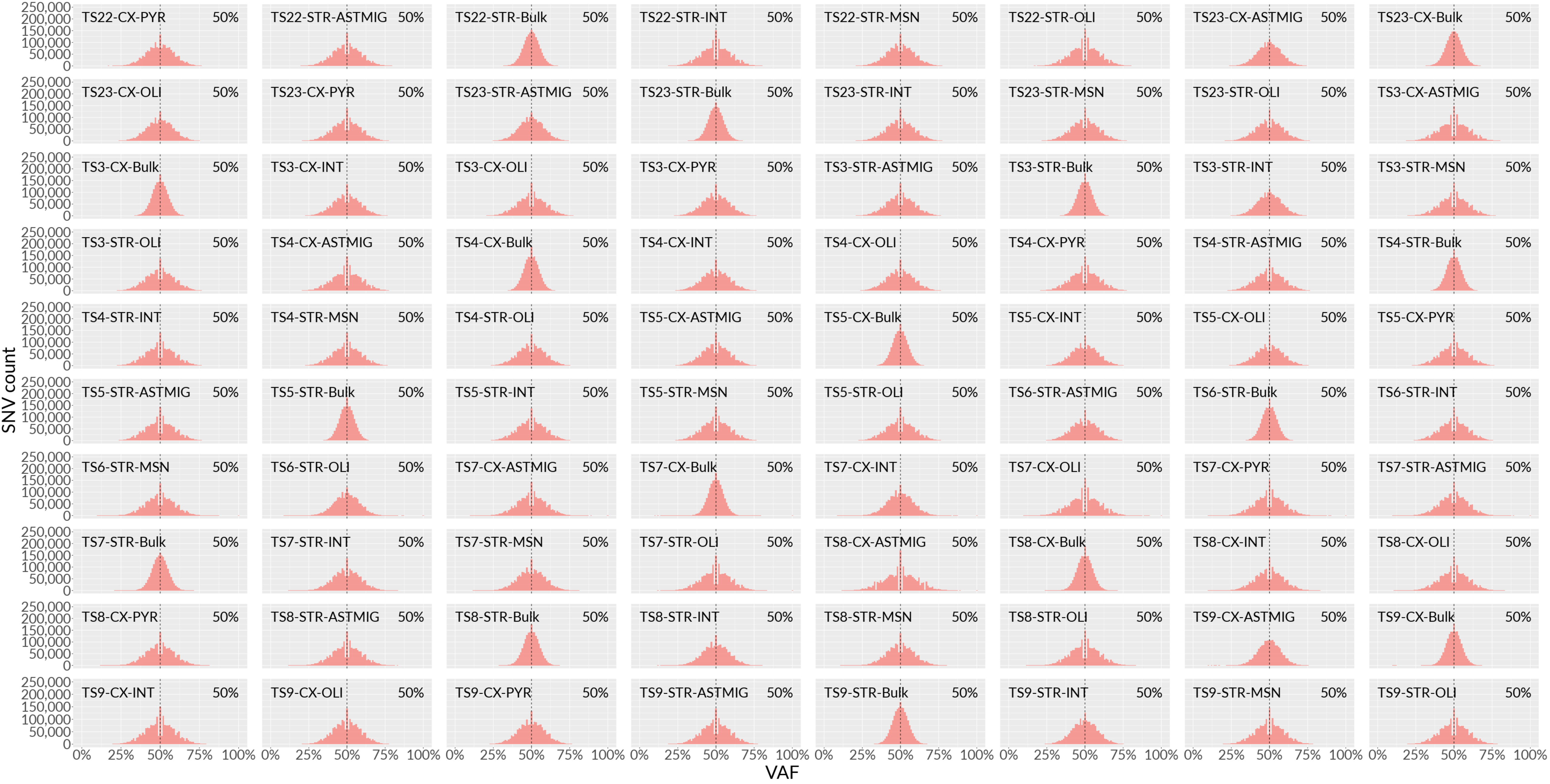

Supp. Fig 3. (e) UCSD (NDA ID 2968) dataset VAF distributions of heterozygous SNPs.

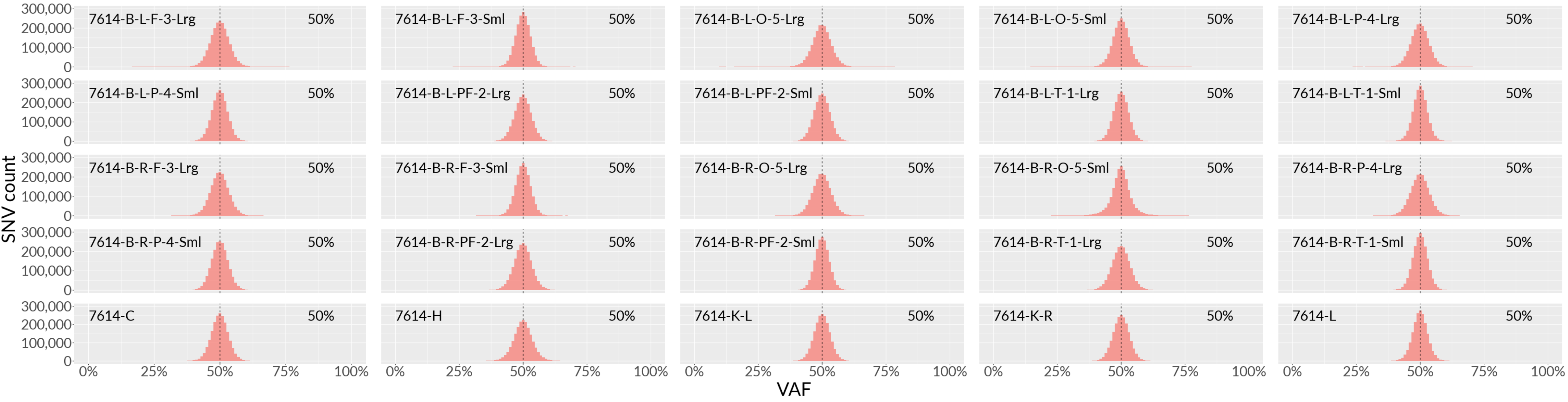

Supplement: Supplementary file 1 — Supplementary figures [file 41597_2023_2645_MOESM1_ESM.pdf]
